# Genome-wide expression profiling of *in vivo-*derived bloodstream parasite stages and dynamic analysis of mRNA alterations during synchronous differentiation in *Trypanosoma brucei*

**DOI:** 10.1186/1471-2164-10-427

**Published:** 2009-09-11

**Authors:** Sarah Kabani, Katelyn Fenn, Alan Ross, Al Ivens, Terry K Smith, Peter Ghazal, Keith Matthews

**Affiliations:** 1 Centre for Immunity, Infection and Evolution, Institute of Immunology and Infection Research, School of Biological Sciences, King's Buildings, University of Edinburgh, Edinburgh, UK; 2 Division of Pathway Medicine, University of Edinburgh Medical School, Chancellor's building, University of Edinburgh, Edinburgh, UK; 3 Fios Genomics Ltd, ETTC, King's buildings, Edinburgh, UK; 4 Centre for Biomolecular Sciences, The North Haugh, St Andrews University, UK

## Abstract

**Background:**

Trypanosomes undergo extensive developmental changes during their complex life cycle. Crucial among these is the transition between slender and stumpy bloodstream forms and, thereafter, the differentiation from stumpy to tsetse-midgut procyclic forms. These developmental events are highly regulated, temporally reproducible and accompanied by expression changes mediated almost exclusively at the post-transcriptional level.

**Results:**

In this study we have examined, by whole-genome microarray analysis, the mRNA abundance of genes in slender and stumpy forms of *T.brucei *AnTat1.1 cells, and also during their synchronous differentiation to procyclic forms. In total, five biological replicates representing the differentiation of matched parasite populations derived from five individual mouse infections were assayed, with RNAs being derived at key biological time points during the time course of their synchronous differentiation to procyclic forms. Importantly, the biological context of these mRNA profiles was established by assaying the coincident cellular events in each population (surface antigen exchange, morphological restructuring, cell cycle re-entry), thereby linking the observed gene expression changes to the well-established framework of trypanosome differentiation.

**Conclusion:**

Using stringent statistical analysis and validation of the derived profiles against experimentally-predicted gene expression and phenotypic changes, we have established the profile of regulated gene expression during these important life-cycle transitions. The highly synchronous nature of differentiation between stumpy and procyclic forms also means that these studies of mRNA profiles are directly relevant to the changes in mRNA abundance within individual cells during this well-characterised developmental transition.

## Background

Gene expression analyses have proved useful for dissecting the basis of the changes that occur as cells and organisms transition between distinct cell types [1-3], developmental stages [4-6] or progress into disease states [7,8]. In many cases, these reflect the regulated activity of gene promoters, or altered stability of mature mRNAs, the integral of these resulting in measurable changes in the steady-state abundance for particular mRNAs. The relative contribution of these two components (regulated synthesis, *vs*. regulated turnover) to changes in the overall transcriptome of a cell varies, although regulated promoter activity has received most attention particularly in the context of development processes and differentiation events [9-11].

In one group of organisms, the balance between mRNA synthesis and turnover shows an extreme emphasis toward regulated mRNA stability. These are kinetoplastid parasites, responsible for an array of diseases in the tropics of medical and veterinary importance [12]. In these organisms, among the most evolutionarily divergent eukaryotes for which there is significant molecular information [13], the contribution of RNA polymerase II promoter activity to regulated gene expression is unimportant [14]. Instead, their genome is organised into long polycistronic transcription units in which genes are co-transcribed [15], primary transcripts being resolved into mRNAs by concerted trans-splicing and polyadenylation reactions [16]. These depend on identifiable RNA processing signals within intergenic regions such that the RNA processing reactions of adjacent genes are mechanistically coupled [17,18]. This arrangement dictates that RNA processing is not a primary regulator of differential expression, since neighbouring genes within a transcription unit often exhibit distinct expression profiles. Hence, regulated mRNA stability is a major contributor to differential mRNA abundance, although regulated protein synthesis, modification and turnover are clearly major additional contributors to regulated gene expression [19,20].

**Figure 1:**
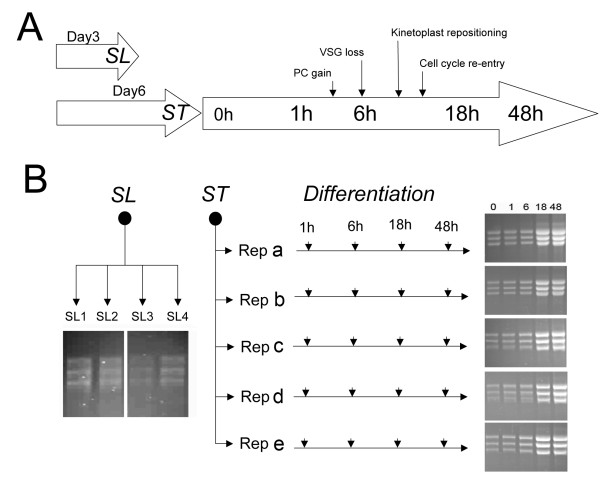
**Schema for the study**. **A**. Schematic diagram of the transitions studied in this investigation. To derive pleomorphic slender (SL) samples, parasitaemias were harvested 3 days post infection. For pleomorphic stumpy (ST) samples, infections were harvested 6 days post infection. From stumpy-enriched samples, differentiation time courses were established, with the major events of this process being annotated, along with their approximate timings. PC= procyclin, VSG = variant surface glycoprotein. **B**. Sample isolations from the experiments carried out in this study. Four bio-replicates of pleomorphic slender forms were derived, SL1, SL2 SL3, SL4. Five independent stumpy form samples were also generated from individual mouse infections (a, b, c, d, e) these being used to initiate five differentiation time courses. RNA samples generated from each bio-replicate are shown, these being those labelled and used for microarray hybridizations.

Although almost exclusively post-transcriptional, differential gene expression is of key importance in kinetoplastid parasites since they undergo complex life-cycles involving transmission between mammalian hosts by arthropod vectors [21,22]. A good model for such developmental transitions is provided by the African trypanosome, *Trypanosoma brucei*. When in the mammalian bloodstream, African trypanosomes exhibit waves of parasitaemia caused by the successive expression of distinct surface antigens by individual 'antigenic variants', these being periodically recognised and destroyed by the host immune response [23]. Superimposed on this cyclical infection profile is a developmental transition induced by quorum-sensing, in which cell-density induces the transition to 'stumpy forms' [24,25]. These differ from the proliferative bloodstream 'slender forms' in that they exhibit cell-cycle arrest in G1/G0 [26], altered morphology [27], and some pre-adaptations for transmission to the tsetse fly such as the up regulation of certain mitochondrial activities [28], and enhanced resistance to protease attack [29] and pH stress [30]. An attractive feature of this developmental step is that stumpy forms accumulate to near homogeneity at the peak of each wave of parasitaemia and can be induced to undergo efficient differentiation to the next life-cycle stage, procyclic forms, if harvested from blood and incubated in culture media at 27°C containing citrate/cis-aconitate (CCA) [31-33]. Importantly, this differentiation is almost completely synchronous in the population allowing events at the individual cell level to be inferred from events occurring at the population level. Moreover, the developmental events of differentiation are reproducible and well characterised such that the progress of cells through differentiation can be accurately monitored [34]. These changes include gain of the procyclic stage-specific coat, comprising procyclins, at 2 h through differentiation, loss of the bloodstream stage-specific variant surface glycoprotein (VSG) coat around 4-7 h [33] and repositioning of the parasite's unusual mitochondrial genome (kinetoplast) between 8 and 14 h through differentiation [35]. Coincident with these changes the cells re-enter into a proliferative cell-cycle, with progression through S-phase occurring between 8 and 12 h [33,36]. Further developmental changes occur after 18 h entailing cell proliferation and metabolic adaptation although these are less synchronous in the population. The general schema of cytological events associated with trypanosome differentiation is presented in Figure 1A.

To date, a number of studies have investigated the differences in mRNA expression profile between cultured procyclic forms and laboratory adapted bloodstream forms, which have lost the ability to generate stumpy forms [37,38]. However, there has been no analysis of the expression profile of mRNAs in pleomorphic slender forms (i.e. those capable of differentiating to stumpy forms), *in vivo *generated stumpy forms or cells undergoing synchronous differentiation from stumpy to procyclic forms. Here we characterise the changes in expressed mRNAs in each of these cellular transitions and place these into the well-characterised cytological framework of differentiation determined using a number of cellular markers. Our experiments emphasise the pre-adaptation of stumpy forms for differentiation and identify novel transcripts enriched in this life-cycle stage or transiently regulated during synchronous differentiation to procyclic forms.

## Results

### Biological sample generation and validation

The biological characteristics of trypanosomes grown in culture likely differ significantly from those in the mammalian bloodstream [38]. Therefore, to generate samples of closest relevance to the *in vivo *situation, slender and stumpy forms were derived from infections in mice. Importantly, our analyses used pleomorphic slender forms, which remain able to generate stumpy forms, unlike previous analyses, which have used laboratory-adapted mutants (monomorphs), which are unable to undergo appropriate growth control either *in vivo *or *in vitro *[37,38]. Since the transition to stumpy forms is induced above ~5 × 10^7 ^trypanosomes/ml, pleomorphic slender forms were harvested at day 3 post-infection and at 2 × 10^7^/ml, a cell density at which no morphologically stumpy forms could be detected. Identical populations were also harvested from mice at day 6 post-infection when the population density was at >2 × 10^8^/ml (the derivation of slender and stumpy forms is summarised in Figure 1A). In these samples, the parasites were overwhelmingly stumpy (>80%) as assessed by morphological criteria. Samples representing the differentiation to procyclic forms were also generated from the same parasite populations that were used to generate stumpy forms. Stumpy forms were purified from host blood by DE52 chromatography [39] at 37°C and then incubated for 1 h at 37°C in HMI-9 [40] to allow the cells to recover from the purification process, this providing the starting material for expression analyses during their differentiation to procyclic forms. Aliquots were harvested to generate stumpy form mRNA and, thereafter, cells were transferred to SDM-79 medium containing 6 mM cis-aconitate and 10 mM glycerol, thus initiating differentiation. Further RNA samples were then isolated at 1 h, 6 h, 18 h, and 48 h after the addition of cis-aconitate, these time points being selected to allow correlation with the major cytological events of differentiation (induction of procyclin cell surface proteins at 1-2 h, VSG loss around 6 h, the generation of early proliferative procyclic forms at 18 h, and more established procyclic forms at 48 h). [41] (Figure 1A). In total four biological replicates of pleomorphic slender cells, 5 biological replicates of stumpy forms and the 5 differentiating populations were isolated (Figure 1B). In each case, total mRNA was purified and its integrity validated both by visualisation on ethidium bromide stained formaldehyde gels (Figure 1B) and via an Agilent RNA 6000 Nano chip using a 2100 Agilent bioanalyser (data not shown).

**Figure 2:**
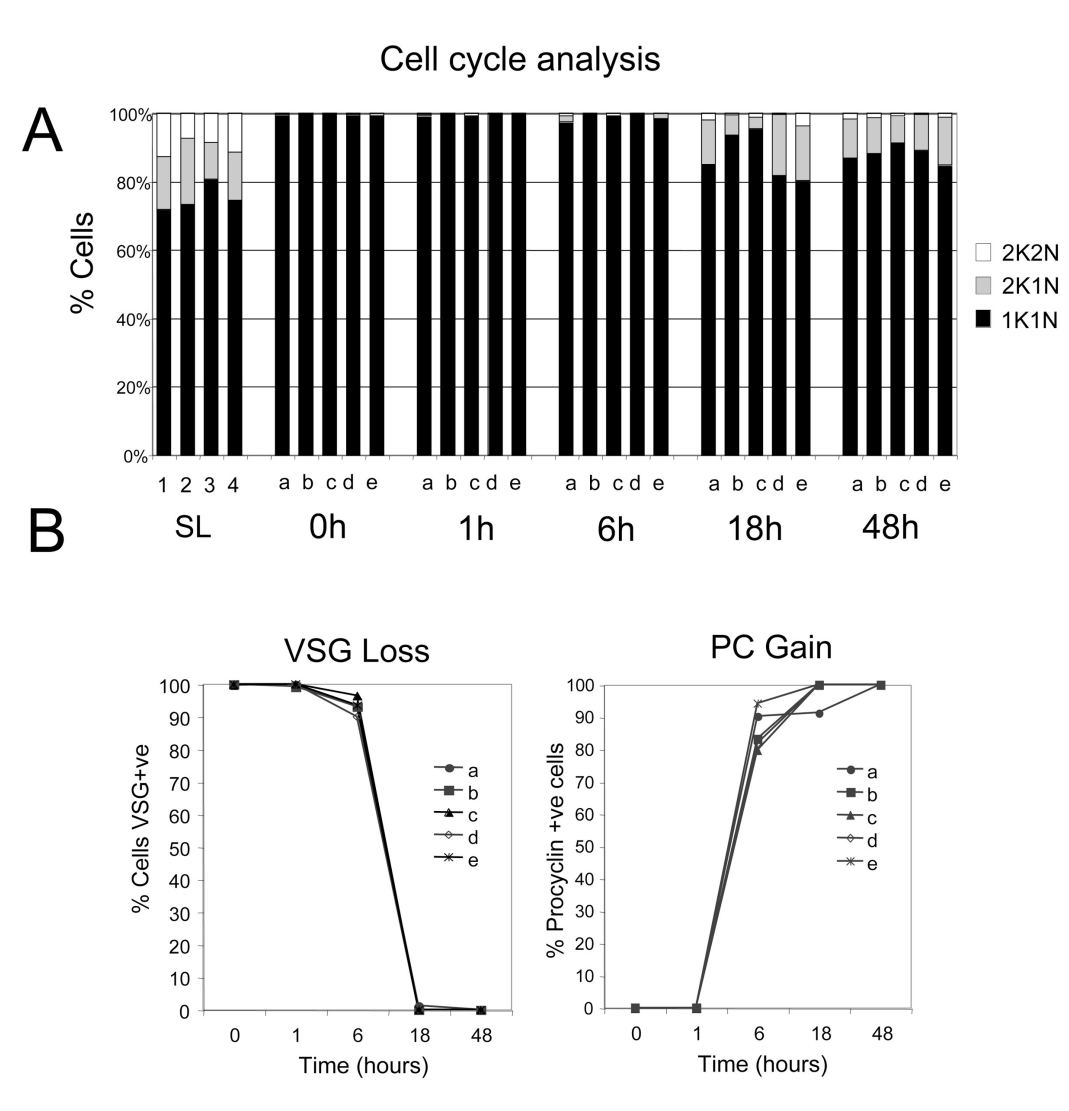
**Cell cycle and surface antigen analysis during differentiation**. **A**. Analysis of the percentage of cells in different cell-cycle phases (1 kinetoplast, 1 nucleus, Black; 2 kinetoplasts, 1 nucleus, grey; 2 kinetoplasts, 2 nuclei, white) from each of the bio-replicates and time points considered in this analysis. Most notably, the stumpy population is uniformly arrested in G1/G0 and cells progressing back into a proliferative cell-cycle begin to appear between 6-18 h after the initiation of differentiation. **B**. Expression of the bloodstream-stage specific variant surface glycoprotein (VSG) coat and EP procyclin (PC) coat on each of the bio-replicates and time points evaluated in this analysis. Samples were scored by immuofluorescence staining for the expression of each antigen. EP procyclin was weakly detectable at 1 h, but strongly expressed within 6 h.

To validate the biological integrity of the material used to generate the mRNAs for array analysis, the trypanosome source material was analysed for their expression of a number of diagnostic cytological markers for development. Firstly, the samples were assayed for cell-cycle progression, this allowing quantitative distinction between proliferative slender and non-proliferative stumpy forms (Figure 2A). In trypanosomes, distinct cell-cycle stages can be distinguished by the co-ordinated replication, and segregation, of the single copy DNA-containing organelles, the nucleus and kinetoplast [42]. Thus cells in G1 (and G0) and S phase exhibit 1 nucleus and 1 kinetoplast (1K1N) whereas G2 phase cells are 2K1N and post mitotic cells are 2K2N. Comparison of the distinct slender and stumpy populations demonstrated that the pleomorphic slender cells were predominantly in the 1K1N configuration (72-81%), but that ~15% (10-19%) of cells were 2K1N and ~10% (7-13%) of cells 2K2N, these values being consistent with the expected profile for proliferative trypanosome populations. In contrast, the stumpy cell populations were enriched for cells in G1/G0 with less than 1% of cells in configurations other than 1K1N (Figure 2A). This, combined with our morphological analysis, confirmed that highly enriched stumpy populations had been generated for each bio-replicate.

Once differentiation is initiated, stumpy cells re-enter into a proliferative cell-cycle with relative synchrony [33]. Although the first morphological event in cell-cycle progression had not occurred in the replicate populations by 1 h or 6 h after the initiation of differentiation, at 18 h cells were seen to be in G2 (2K1N; 3-18%) or in the mitotic/post mitotic phase (2K2N; 0.5-4%), with similar proportions seen at 48 h. This indicated that the cells had progressed from cell-cycle arrest into a proliferative cell-cycle during the time course of differentiation.

In addition to cell-cycle progression the differentiating cells were also assayed for their expression of the stage-specific surface antigens, VSG (expressed on bloodstream forms) and EP procyclin (induced during differentiation and expressed on procyclic forms) (Figure 2B). Matching a normal profile of differentiation, both slender and stumpy bloodstream populations expressed the AnTat1.1 VSG, as did cells during the first 6 h of differentiation. However, thereafter, VSG was no longer detected excepting on a small number of undifferentiated cells, these representing the small proportion of proliferative slender cells present in the stumpy-enriched population [33]. The expression of EP procyclin differed from VSG during differentiation, such that it was already detectable 1 h after the addition of cis-aconitate, although this was relatively weak and restricted to the flagellar pocket region. By 6 h however, when the cells retained VSG, EP procyclin was strongly expressed over the cell surface of the differentiating parasites (79-94%) and this was retained in the 18 h and 48 h samples, as expected.

**Figure 3:**
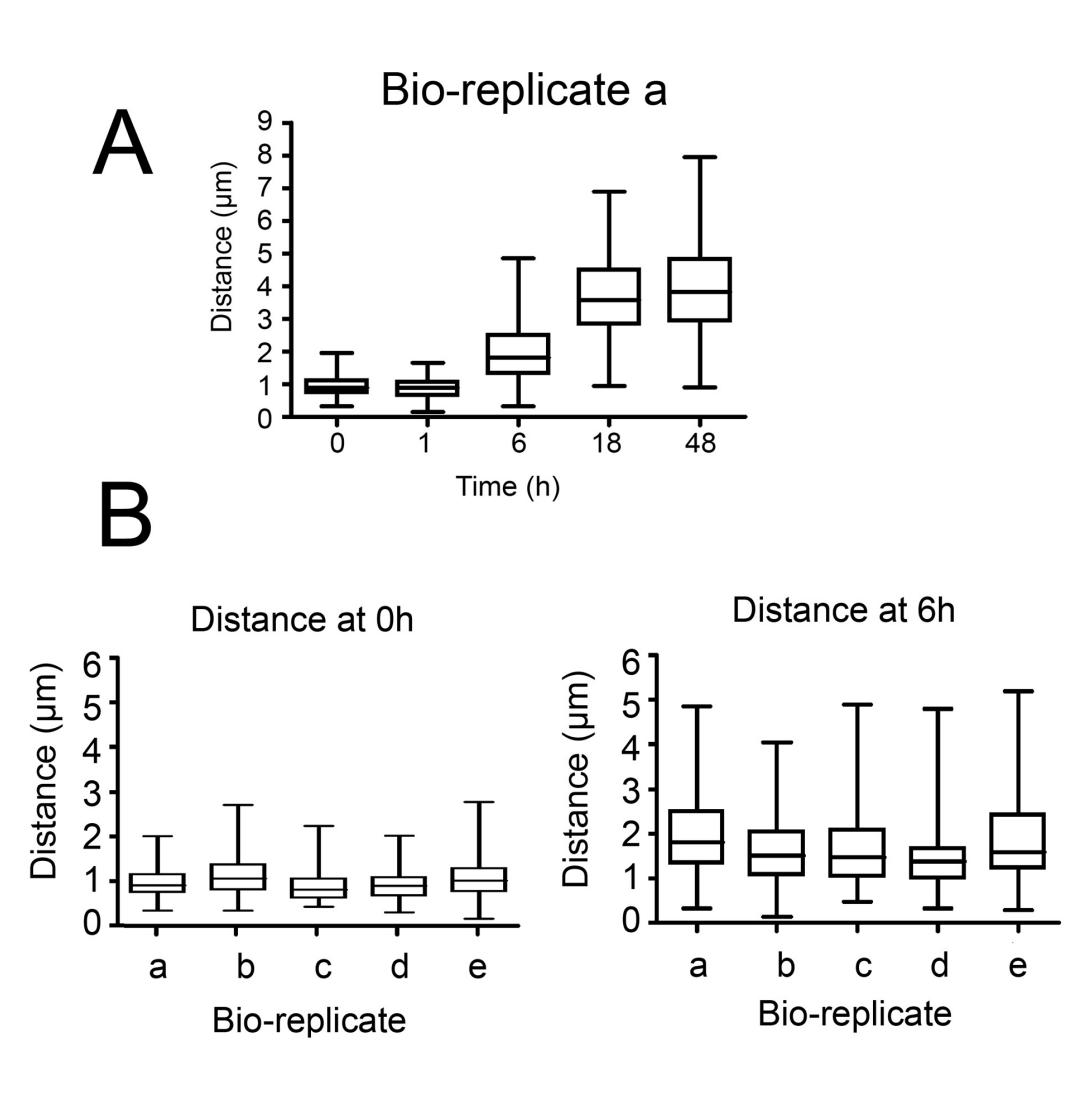
**Kinetoplast repositioning during differentiation**. **A**. Repositioning of the kinetoplast for cells undergoing differentiation from stumpy forms. Values represent the distance from the kinetoplast to the cell posterior measured for 100 cells at each time point and are derived from bio-replicate a. **B**. Repositioning of the kinetoplast for all bio-replicates, measured at 0 h and 6 h after the initiation of differentiation. Measures represent the distance between the kinetoplast and cell posterior for 100 cells at each time point and for each bioreplicate.

The final measure we used to monitor the progression of cells through differentiation was repositioning of the mitochondrial genome. During trypanosome differentiation, the kinetoplast is relocated from the extreme posterior of bloodstream form cells to midway between the cell nucleus and cell posterior in procyclic forms [35]. Analysis of 100 cells from each time point of one complete differentiation series (bio-replicate a) demonstrated that kinetoplast repositioning had initiated after 6 h, with this then continuing until 18 h, when the kinetoplast-posterior dimension had increased from ~1 μm to ~4 μm (Figure 3A). Analysis of the kinetoplast-posterior dimension of 100 cells in each of the remaining four bio-replicates at 1 h and 6 h confirmed that all the samples used for expression analysis repositioned their kinetoplast on approximately the same timescale, with the initiation of differentiation being detected by 6 h (Figure 3B).

Combined, these assays provided the essential cytological framework necessary for interpretation of the expression profile of mRNAs from each of the derived samples. Most significantly, the 5 biological replicates of the populations undergoing differentiation from stumpy to procyclic forms underwent differentiation with reproducible kinetics despite their derivation from 5 independent rodent infections and differentiation assays. Hence, the derived material provided a robust and biologically relevant dataset for the analysis of mRNAs regulated during the associated differentiation events.

### Microarray expression profiling of the trypanosome cells in different life-cycle forms or stages of development

**Figure 4:**
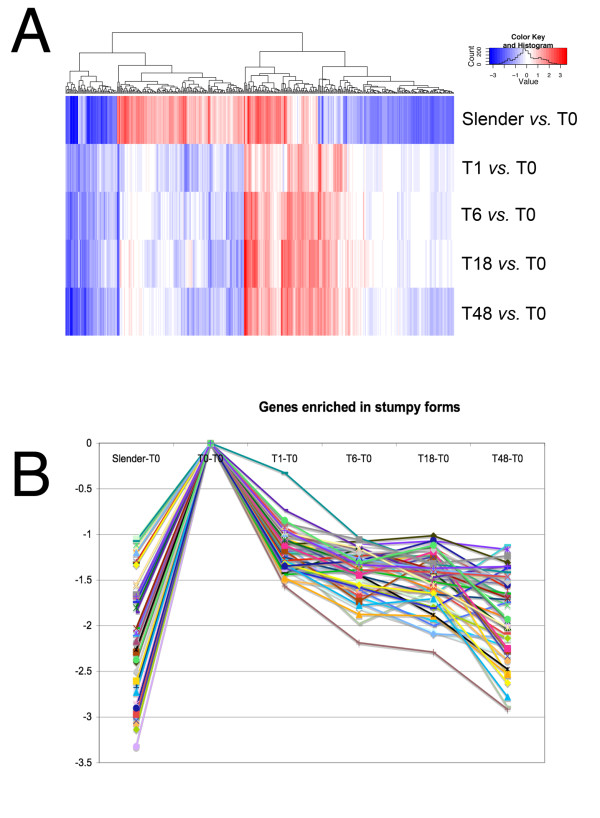
**Expression profiles of genes at different time points in the analysis**. **A**. Heatmap of expression differences of all samples relative to stumpy samples (T = 0). The genes included were significant at the p < 0.001 level in one or more contrasts. The logFC values have been heatmapped, with blue representing down-regulation and red representing up-regulation. Genes are along the x-axis and comparisons are represented along the y-axis, with gene profiles being clustered by Euclidian distance, their relatedness being shown in the dendogram above the heatmap. **B**. Expression profile of genes identified as being enriched in stumpy forms. The genes shown are those identified in the trinary groups (-1,-1,-1,-1,-1) and (0,-1,-1,-1,-1). The y axis represents the LC fold change, the X-axis is the time point during the differentiation programme.

In total, 29 RNA samples were generated (5 biological replicates of stumpy, 1 h, 6 h, 18 h and 48 h; 4 biological replicates of slender) and hybridized to JCVI *Trypanosoma brucei *v3 arrays, comprising 19,200 features representing 8,801 different loci, of which 8,300 were *T. bruce*i genes. The resulting hybridization profiles were assessed pre- and post-quantile normalization and from this analysis four arrays were identified as being sub-standard and subsequently removed from the analysis. Thereafter, a design matrix was established in order to describe the array hybridizations with respect to the sample time point, with bio-replicates being combined to generate group comparisons. In total 12 group comparisons were generated, detailing 'slender compared to all others', 'stumpy compared to all others' in addition to comparisons through a moving window throughout the differentiation time course (i.e. T1 *vs*. T6, T6 *vs*. T18 and T18 *vs*. T48). For these analyses, normalised data were linear model fitted and Empirical Bayesian analysis, coupled with p value adjustment, performed (full comparison data is available in Additional file 1 and Additional file 2). From the resulting comparisons, a total of 407 genes were identified which exhibited at least 2-fold change in at least one comparison, with an associated p value of <0.001 (Additional file 3 shows the expression profile of this 407 gene set; the expression profile of all genes irrespective of their significance is also included in the same folder). From the 407 gene set, it was clear that the distinctions between slender and all other samples were greater than the distinctions between the samples derived from within the differentiation time course (Figure 4). This was not surprising for two reasons. Firstly, the distinct time-points from the differentiation samples were biologically linked, such that each time point sample from each bio-replicate was derived from a common progenitor (see Figure 1B). Hence the variation within these sample series is expected to be relatively low compared with comparisons from independent samples. Secondly, stumpy cells had already undergone significant changes in expression compared to slender cells, likely reflecting their pre-adaptations for differentiation and possible embarkation on some aspects of the differentiation programme during their isolation and purification from blood. Although the purification from blood was carried out rapidly at 37°C, stresses resulting from the isolation procedures could have induced these early changes in expression. Hence, both stumpy-enriched and early procyclic form transcripts would be expected in these 'stumpy' mRNA profiles.

### Analyses of transcripts up-regulated in stumpy cells

Using a stringent threshold of an adjusted P value of < 0.05 for comparison, 42 genes were scored as up-regulated and 41 were scored as down-regulated when comparing stumpy forms with slender forms (Additional file 4). At a slightly reduced stringency of an adjusted P value of < 0.1, 96 genes were scored as 'up in slender' whereas 190 genes were 'up in stumpy' (Additional file 4). Consistent with expectation, slender cells expressed significantly more ESAG transcripts (ESAG2, ESAG11, ESAG5) reflective of the down-regulation of VSG expression site transcription in stumpy forms [43]. Also, histone transcripts (histone H2, H3, H4) and an HMG protein (Tb927.3.3490), were elevated in slender forms as were mRNAs for structural components of the cell (e.g. PFR1, PFR2, beta-tubulin) and components of the translational apparatus (Tb927.8.5880, Tb11.01.5720, Tb11.02.4050, Tb927.5.1610). These changes reflect the proliferative status of slender forms compared to the cellular quiescence of stumpy cells [44]. The metabolism of slender forms was also represented by the enrichment of the bloodstream form glucose transporter THT1 (Tb10.6k15.2040) [45], aldolase [46] and PGKC (Tb927.1.700) [47].

Transcripts enriched in stumpy forms when compared to slender forms (at an adjusted P value of < 0.05) included the mRNA for the procyclic surface protein EP3 procyclin (Tb927.6.480), membrane protein and lipid biosynthesis genes (Tb09.211.1030; Tb10.6k15.3610), a chloride channel protein (Tb09.211.0430) and a number of translation and RNA binding proteins including a gene encoding deoxyhypusine synthase activity (Tb10.70.4900; required for elongation factor 5A modification and cell proliferation), Ribosomal protein s10 (Tb10.70.1690; required for tRNA association with the ribosome and transcription elongation), an elongation factor of the Tu factor family (Tb11.03.0940; a GTP binding elongation factor) and an ATP-dependent RNA helicase (Tb10.70.6180). Of these enriched mRNAs, Tb09.211.1030 has been recently characterised as *TbSLS1*, a protein involved in phosphosphingolipid synthesis [48] (see later), whereas Tb09.211.0430 is a homologue of the CLC7 family of lysosomal chloride channel proteins. These may be involved in the maintenance of lysosomal pH balance in stumpy forms, where lysosomal activity is considerably enhanced [49,50], Jay Bangs, personal communication). Finally, we observed that in the enriched set was one member of the PAD array family of proteins (PAD6; Tb927.7.5980) that have recently been identified to be involved in citrate/cis-aconitate mediated differentiation in stumpy forms parasites under cold shock conditions [51]. The absence of PAD1 (Tb927.7.5930), recently identified as being up-regulated in stumpy forms at the mRNA and protein level [51], reflects the stringency of the threshold used, with this gene being detected, along with PAD 6 (Tb927.7.5980) and PAD8 (Tb927.7.6000) when the slightly reduced stringency was applied (adj P value < 0.1) (Additional file 4).

The presence of abundant procyclin and procyclic-enriched mRNAs in the stumpy form mRNA samples indicated that either these cells exhibit pre-adaptation for differentiation at the mRNA level, or that some of these mRNAs were rapidly induced during the parasite isolation procedures. Therefore, to identify transcripts likely to be enriched in stumpy forms, transcript expression profiles from all time points were assigned to a trinary scoring system, where transcript levels were expressed relative to their level in stumpy forms. Hence, transcripts with no significant change (less than 2-fold change, P > 0.05) would be annotated 0,0,0,0,0 (1 h *vs*. ST, 6 h *vs*. ST, 18 h *vs*. ST, 48 h *vs*. ST, SL *vs*. ST,) whereas transcripts up-regulated in stumpy forms (>2-fold change, p < 0.05) with respect to all other time points would be annotated -1, -1, -1, -1, -1. This group, along with transcripts also elevated at t = 1 h (0, -1, -1, -1, -1), was assigned as "stumpy -enriched" and comprised a total of 65 genes. The full list of these genes is available in Additional file 5, and those predicted to encode functional proteins are summarised in Table 1. The predicted stumpy-specific group comprised mRNAs encoding two RNA helicases (Tb10.70.6180, Tb09.211.2300), an additional chloride ion channel protein (Tb10.26.0220), a PAD member (Tb927.7.6000; PAD8), enzymes involved in membrane sphingolipid (Tb927.4.1020; a putative serine-palmitoyl-CoA transferase) and sterol biosynthesis (Tb11.02.0780; a putative squalene monooxygenase) and the metabolic enzymes trypanosome alternative oxidase and fructose-2, 6-biphosphatase. The enzyme MSP-B, elevated early in differentiation and associated with VSG release in this process, was also elevated in the stumpy sample and through differentiation when compared with more established procyclic forms, this matching previous observations [52]. Widening the set to include transcripts elevated in stumpy cells and cells at 1 h, included two zinc-finger proteins, one of the CCCH class (Tb927.6.4960), members of which have previously been demonstrated to be important in differentiation control [53-56]. In common with these proteins, Tb927.6.4960 has only a single CCCH domain, which has closest similarity to members of the OMA family of proteins involved in *C.elegans *oocyte maturation [57]. Other proteins included a dual specificity phosphatase (Tb927.7.7160), a calcium pump protein (Tb927.5.3400) and a recently characterised nucleobase transporter (Tb09.244.2020; NT11 [58]).

**Table 1:** Transcripts with expression profile: -1,-1,-1,-1,-1

**Gene ID**	**Product**	**Notes**
Tb09.211.0460	hypothetical protein, conserved	
Tb11.02.2180	hypothetical protein	GPI anchor
Tb927.6.4180	hypothetical protein, conserved	
Tb927.3.5770	hypothetical protein	
Tb11.01.0470	hypothetical protein, conserved	
Tb10.70.1690	40S ribosomal protein S10, putative	Ribosome
Tb10.6k15.3640	alternative oxidase	TAO
Tb927.3.2540	variant surface glycoprotein related, putative	
Tb927.7.3170	hypothetical protein, conserved	
Tb10.6k15.0300	hypothetical protein, conserved	
Tb10.389.0650	hypothetical protein, conserved	
Tb11.47.0019	hypothetical protein, conserved	membrane
Tb927.4.1020	serine-palmitoyl-CoA transferase, putative	
Tb927.8.1270	hypothetical protein, conserved	
Tb11.02.0780	squalene monooxygenase, putative	
Tb927.8.1610	major surface protease gp63, putative, GP63, putative, metallopeptidase, putative	MSP-B
Tb10.70.6180	ATP-dependent DEAD/H RNA helicase, putative	RNA helicase
Tb927.7.6000	hypothetical protein, conserved	PAD8
Tb927.8.6800	hypothetical protein, conserved	
Tb927.5.2320	hypothetical protein, conserved	
Tb09.160.3060	hypothetical protein	
Tb09.211.2300	ATP-dependent DEAD/H RNA helicase, putative	RNA helicase
Tb927.5.4110	hypothetical protein, conserved	
Tb10.61.0450	hypothetical protein, conserved	
Tb927.7.3980	immunodominant antigen, putative, tc40 antigen-like	Tc40 antigen-like
Tb10.26.0220	chloride channel protein, putative	
Tb927.3.2710	6-phosphofructo-2-kinase/fructose-2,6-biphosphatase, putative	
Tb927.1.4450	hypothetical protein, conserved	
Tb10.61.3040	hypothetical protein, conserved	
		
**Transcripts with expression profile: 0,-1,-1,-1,-1**		
**Gene ID**	**Product**	**Notes**
Tb11.02.0370	hypothetical protein, conserved	GPI anchor
Tb927.8.5670	hypothetical protein, conserved	
Tb10.70.4310	hypothetical protein	
Tb11.02.5260	hypothetical protein, conserved, predicted zinc finger protein	Zn Finger
Tb927.6.4960	zinc finger-domain protein, putative	CCCH Zn finger
Tb11.55.0005	hypothetical protein, conserved	
Tb09.244.2020	nucleoside transporter 1, putative	NT11
Tb927.5.1760	hypothetical protein, conserved	
Tb927.7.7320	hypothetical protein, conserved	
Tb10.6k15.3300	hypothetical protein, conserved	
Tb927.7.7160	dual specificity protein phosphatase, putative	Dual specificity Phosphatase
Tb927.5.3400	calcium-translocating P-type ATPase, calcium pump	Calcium pump protein
Tb927.2.4950	hypothetical protein, conserved	

Genes predicted to encode protein products and whose mRNA expression profile was elevated in stumpy forms with respect to slender forms or during synchronous differentiation to procyclic forms. Genes specifically elevated in stumpy forms only, and genes elevated in stumpy forms and 1 h after the initiation of differentiation to procyclic forms, are shown.

In addition to those known genes, or genes for proteins with predicted function, were a group of genes of no predicted function, being annotated hypothetical unlikely. This group, comprising 25% of the stumpy-enriched mRNA representatives (Additional file 5), were unexpected and among the transcripts most highly enriched in stumpy forms when compared to slender forms. These genes were frequently small, or positioned on the opposite strand to the predicted polycistronic transcription unit. The presence of these transcripts may represent dysregulated control of genome expression in quiescent stumpy forms, non-specific transcriptional noise or a hitherto unrecognised feature of gene expression or regulation in trypanosomes.

### Phenotypic correlation of transcript profiles

**Figure 5:**
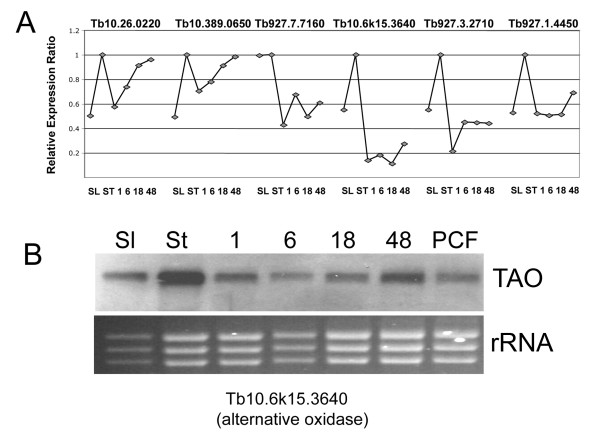
**Validation of the microarray by alternative expression assays**. **A**. qRT-PCR analysis of the expression profile of six test transcripts identified as being enriched in stumpy forms by microarray hybridization. The relative expression is shown (y-axis) at each time point (x-axis), the expression ratio being normalised to a control transcript (Tb10.389.0540). **B**. Northern blot showing the relative expression of trypanosome alternative oxidase (Tb10.6k15.3640) in samples from one time course assay, using the same mRNAs used for microarray hybridization. The relative loading is indicated by the rRNA beneath the blot, this being revealed by ethidium bromide staining.

To assess the validity of the observed expression profiles for stumpy elevated transcripts, a subset of 6 genes from the 'stumpy-enriched' (-1, -1, -1, -1, -1) trinary group and a control transcript (Tb10.389.0540), which was well expressed but had low standard deviation across all time points, were evaluated by qRT-PCR against the mRNA samples used to hybridize to the array replicates. Figure 5A demonstrates that each transcript observed to be up-regulated in stumpy forms by microarray was confirmed as being elevated at that life-cycle stage by qRT-PCR, although the anticipated expression levels did not precisely correspond with the array profile. In addition, analysis of one transcript predicted to be stumpy-enriched (TAO) was confirmed by northern blotting (Figure 5B). However, four other transcripts (Tb10.70.6180, Tb927.5.3400, Tb927.8.1270, Tb927.7.3170) analysed by qRT-PCR did not clearly correspond to the array data. Analysis of the raw array hybridization data for these transcripts revealed consistent hybridization profiles between duplicate spots on individual arrays and between distinct bio-replicates, these matching the expression trends of the group normalised data. Hence, observed discrepancies may reflect the inability to precisely model array normalisations by assays on individual mRNA samples in our validation experiments, differences in primer or probe recognition sites or non systematic errors.

**Figure 6:**
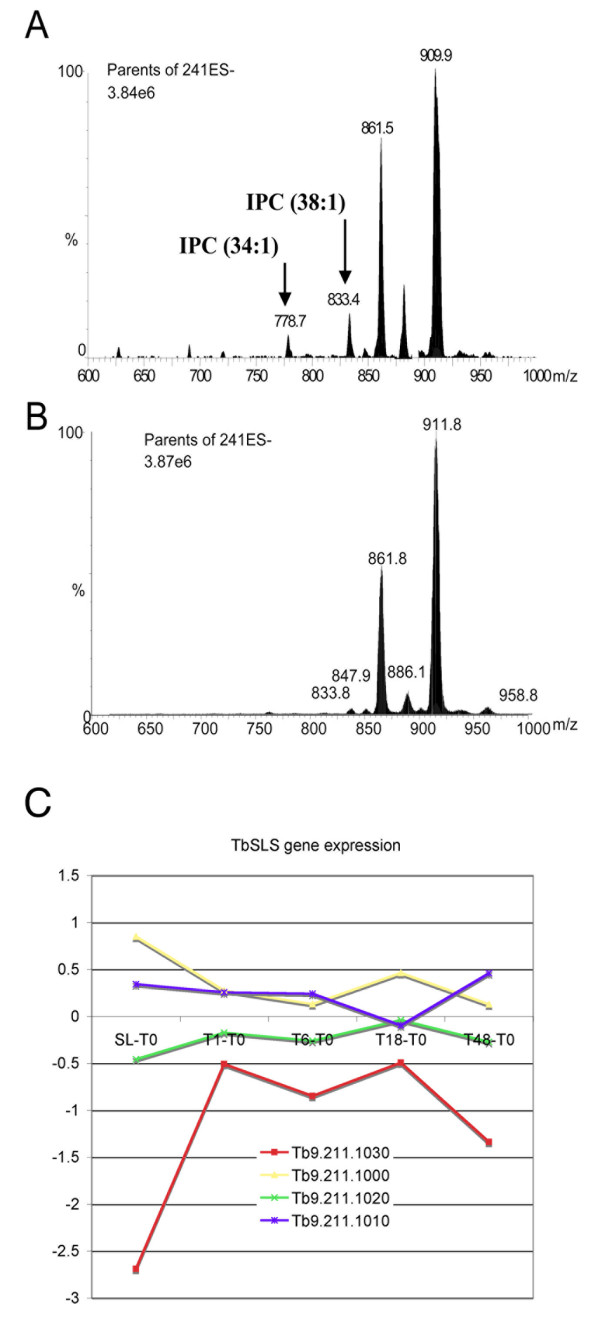
**IPC expression in different life cycle stages**. Lipids extracted from stumpy (**Panel A**) and monomorphic slender (**Panel B**) *T.brucei*, were analyzed by ES-MS/MS for GPIno phospholipids by parent-ion scanning of the m/z 241 as described in experimental procedures. Peak assignments are based on MS/MS daughter ion spectra and comparisons to previous experiments on whole cell extracts. **Panel C **shows the relative expression of each member of the *TbSLS *gene family (*TbSLS1*, Tb9.211.1030; *TbSLS2*, Tb9.211.1020; *TbSLS3*, Tb9.211.1010; *TbSLS4*, Tb9.211.1000) during the differentiation between slender and stumpy forms and at each point during differentiation from stumpy to procyclic forms. *TbSLS1 *is significantly up-regulated during these transitions.

To investigate cytological predictions implicated by the microarray data, a phenotypic analysis of the array data was performed based on the observed up regulation in stumpy vs. slender samples (p < 0.05; Additional file 4) of a predicted phosphatidylcholine: ceramide cholinephosphotransferase (sphingomyelin synthase). This is the first gene in a cluster of four related genes, *TbSLS1-4*, responsible for the synthesis of trypanosome phosphosphingolipids [48]. In *T. brucei*, sphingolipid synthesis is developmentally regulated, with inositol phosphoceramide (IPC) being produced by procyclic, but not bloodstream monomorphic slender forms [48]. To determine whether Tb09.211.1030 expression correlated with the appearance of IPC in stumpy forms, monomorphic slender and pleomorphic stumpy *T. brucei *phospholipids were analysed by ESI-MS. Since comparisons of the negative and positive ion survey scans showed no substantial differences between each cell type (data not shown), a more detailed investigation by ESI-MS-MS using parent ion scanning of individual phospholipid classes by specific collision induced fragmentation was carried out [59]. This revealed that only the phosphatidylinositol (GPIno) phospholipids were different between slender and stumpy forms (compare Figures 6A and 6B), the most telling of which were the peaks at 778.7 m/z and 833.4 m/z (Figure 6A). These correspond to two IPC species (C34:1 and C38:1, respectively), which were detected only in stumpy forms (Figure 6A). This demonstrates that the induction of IPC synthesis, either in stumpy forms or as a very early event in their differentiation to procyclic forms, is indeed coincident with the up-regulation of Tb09.211.1030. Interestingly, analysing the expression profile of each member of the *TbSLS *gene family indicated that only Tb09.211.1030 (*TbSLS1*) showed significant developmental up-regulation (Figure 6C). Therefore, this analysis provided a phenotypic validation of expression profiles identified by microarray analysis and also suggested that the developmental regulation of IPC synthesis may depend on the regulated expression of Tb09.211.1030 (*TbSLS1*) rather than the activity of the other genes in this cluster. This prediction has been recently confirmed providing functional support for the expression changes we observed (Jay Bangs, University of Wisconsin, USA, personal communication).

### Trends of expression during synchronous differentiation from stumpy to procyclic forms

**Figure 7:**
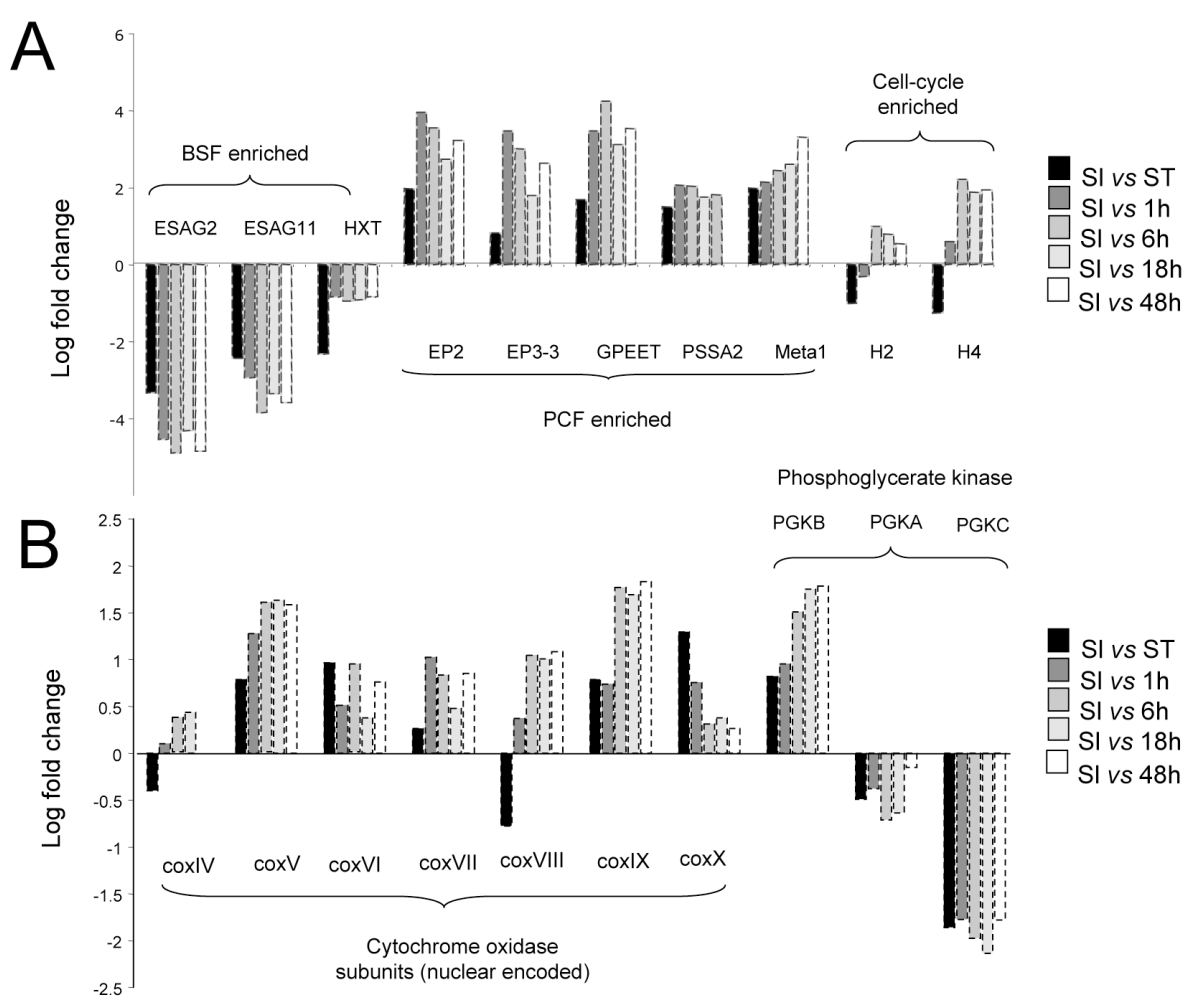
**Expression changes for a subset of genes whose developmental expression profile is known**. **A**. Relative expression of various transcripts known to be regulated between bloodstream monomorphic forms and procyclic forms. The expression of each transcript is represented such that they are expressed relative to their level in pleomorphic slender forms (this being normalised to zero). Hence, each transcript begins with a 'zero-value' column, representing the slender *vs*. slender comparison. ESAG 2, ESAG11 and the glucose transporter HXT (THT1) are bloodstream enriched, whereas the transcripts encoding EP2, EP3-3 and GPEET procyclin, PSSA2 and a molecule related to the *Leishmania *metacyclic-promoastigote Meta 1 protein are procyclic enriched. Cell cycle regulated transcripts; histone H2a and histone H4 are induced around 6 h after the initiation of differentiation, coincident with preparation of the cells for cell-cycle re-entry (Figure 2A). **B**. Relative expression of proteins associated with metabolic development of parasites as they differentiate to procyclic forms. Seven nuclear encoded components of the cytochrome oxidase complex have been previously characterized with respect to their developmental expression and control. Also shown is the relative expression of PGK A, PGK B and PGK C which are differentially regulated in the trypanosome life cycle. All transcripts show the expected expression profiles. Values are expressed relative to the expression of each transcript in pleomorphic slender forms.

In addition to comparisons of slender and stumpy forms, we also exploited the synchrony of differentiation between stumpy forms and procyclic forms to examine transcripts transiently regulated during the differentiation programme. Initially we analysed the expression profile of known transcripts predicted to exhibit temporal or differential regulation during the development from bloodstream to procyclic forms. This involved analysis of the expression profile of stage-specific surface proteins expressed in bloodstream and procyclic forms, markers for re-entry into a proliferative cell-cycle and transcripts associated with the differential metabolism of bloodstream and procyclic forms. Figure 7 shows the expression of 20 transcripts throughout the differentiation time course, with each exhibiting the expected expression profile. Thus, ESAG2, ESAG11 and the glucose transporter THT1 (Tb10.6k15.2040) were rapidly and progressively down-regulated during differentiation, whereas EP2 and EP3 procyclin transcripts were up-regulated, as was PSSA2 [60] and a homologue of the *Leishmania *metacyclic-promastigote expressed virulence protein Meta1 (Tb927.5.2160; [61]). Also, re-entry into DNA synthesis by the differentiating parasites was reflected by the induction of histone mRNAs after 6 h, this immediately preceding detectable progression of the cells into a cell-cycle as determined in our DAPI-scoring of the parasites from which the mRNA samples were derived (Figure 2) [33]. The metabolic adaptation of the differentiating parasites was also confirmed by the differential mRNA regulation of phosphoglycerate kinase C, which was down-regulated, and the up regulation of the procyclic form specific PGKB [47]. The mRNA abundance of the constitutively expressed PGKA was not altered significantly during the differentiation programme, as expected. Finally, we assayed the mRNA expression profile of seven nuclear encoded components of the cytochrome oxidase complex, which are known to be induced at the mRNA level and have previously been characterised during the synchronous differentiation of stumpy forms to procyclic forms [62]. Confirming earlier analyses, each subunit was up-regulated during differentiation to the procyclic form, this occurring rapidly after the initiation of the process. Combined, these analyses demonstrated that the mRNAs generated from parasites undergoing synchronous differentiation from stumpy to procyclic forms, matched expectation based on known bloodstream *vs*. procyclic expression profiles or previous studies of synchronous differentiation between bloodstream stumpy and procyclic forms.

**Figure 8:**
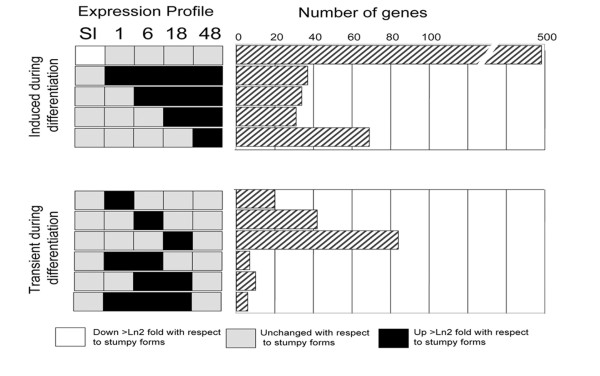
**Dynamic expression profile changes during differentiation between stumpy and procyclic forms**. The expression profile of each transcript cohort is shown on the left hand side and represents the overall expression of transcripts relative to stumpy forms. Transcripts induced at different times during differentiation are shown on the top panel, whereas transcripts transiently elevated during development are shown on the bottom panel. The right hand side shows the number of genes in each cohort.

Having carried out a validation of the observed dynamic mRNA changes during differentiation from stumpy to procyclic forms, we examined the regulated mRNA populations for temporally-regulated changes that were detectable during the synchronous differentiation programme. As before, a trinary scoring system was used to identify the expression profiles of transcripts sets, which exhibited up-, or down-regulation (>2-fold change, p < 0.05) at distinct time points. Of the total of 243 distinct profiles possible, 74 distinct expression profiles were detected. However, most of these differences represented distinctions between the slender and stumpy forms, and 15 of the groups contained only a single member (data not shown). Figure 8 shows a representation of eleven of the most relevant different expression profiles in which transcripts were up-regulated with respect to stumpy forms after 1 h, 6 h, 18 h and 48 h or were transiently up-regulated at each time point (1 h, 6 h, 18 h) or during a combination of time points (1 h and 6 h; 6 h and 18 h; 1, 6, and 18 h). A summary of these transiently regulated mRNAs is available in Additional file 6, whereas a complete expression profile of all genes showing transiently regulated changes is provided in Additional file 7. As expected the greatest difference was between slender and stumpy forms (Figure 8). However transcript groups with distinct expression profiles during the time course of differentiation were also observed. In particular, among those transcripts transiently elevated during distinct phases of the differentiation time course were nucleoside and amino acid membrane transporters (Tb927.8.7670, Tb927.4.4000, Tb11.02.1100; each elevated at t = 1 h) and a predicted serine threonine phosphatase (Tb09.160.4460; elevated at 1 h) identified as a substrate of the differentiation regulator, TbPTP1 (Szöor et al., manuscript in preparation). The activation of translation early in the differentiation time course was also evidenced by the enrichment of mRNAs for two nucleolar proteins (NOP44/46 [63], elevated at 1 h and 6 h), a nucleolar RNA helicase (Tb927.5.4420, elevated at 1-18 h), and ribosomal components (Tb10.70.5670, EIF1α).

**Figure 9:**
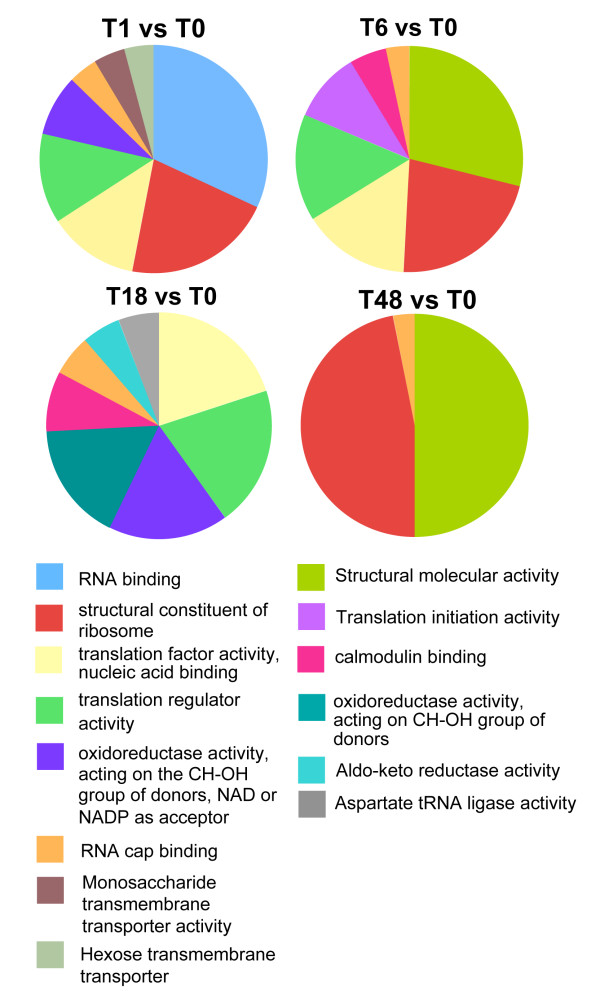
**Gene ontology classifications for transcripts elevated at different time points after the initiation of differentiation from stumpy forms to procyclic forms**. Up-regulated genes at each time point were classified according to the 'molecular function' gene ontology and the frequency of members of each classification plotted as a fraction of the total number of regulated genes. The source data for genes up- and down- regulated during differentiation and at transient windows throughout the differentiation programme are provided in Additional file 8.

In order to functionally classify transcripts during the differentiation events, a GO analysis of the significantly regulated mRNAs was performed. Thus, transcripts significantly regulated at the P < 0.05 level were analysed across the three GO ontologies (biological process, molecular function, cellular compartment) using hypergeometric tests, with enrichment of any one GO group (P < 0.01) being scored independently for genes up and down-regulated in each comparison with respect to the GO-group frequency for all genes on the array. The complete list of GO assignments of those genes up- or down- regulated during differentiation from stumpy to procyclic forms is provided in Additional file 8. Analysis of those genes whose expression was temporally regulated during differentiation demonstrated that upon the initiation of differentiation to procyclic forms (t = 1 h), transcripts associated with RNA translation, transport and regulation were rapidly enriched, whereas endonucleases were down-regulated, this matching the overall increase in mRNA abundance and protein synthesis predicted as the cells enter into the differentiation programme (Figure 1B, [64]). Within 6 h of the initiation of development to procyclic forms, translation associated mRNAs (EIF4E, Tb10.61.0210) were enriched with respect to T = 1 h, as were mRNAs encoding proteins associated with DNA replication (histone H2A, Tb927.7.2870; histone H2B, Tb10.406.0370; histone H4, Tb927.5.4240, histone deacetylase 2, Tb11.01.7240; a minichromosome maintenance protein complex subunit, Tb927.2.3930 and an HMG protein, Tb927.3.349) and structural proteins (PFRA, Tb927.3.4310; PFRB, Tb927.8.4990; flagellum calcium binding protein, Tb927.8.5460; alpha-tubulin, Tb927.1.2360 and beta-tubulin, Tb927.1.2370). These are each consistent with preparation of the differentiating cells for re-entry into the proliferative cell-cycle, which occurs at 8-10 h into the differentiation programme. Although there were relatively few transient changes in mRNAs with discrete GO classification groups beyond 6 h, there was a significant and progressive trend for the up-regulation of genes contributing to translation, and trans splicing during the differentiation programme, matching expectation as stumpy forms progress from translational quiescence into proliferative procyclic forms which are highly active in protein synthesis with abundant polyribosomes [44]. A summary of the GO groups elevated at each time point with respect to stumpy cells (assigned according to their frequency in each 'molecular function' category) is presented in Figure 9, whereas a profile of the statistically significant changes in GO group representation during differentiation is shown in Table 2.

**Table 2:** Enrichment or reduction of GO groups with respect to stumpy forms

**Comparison**	**Enriched with respect to Stumpy**	**Reduced with respect to Stumpy**
**Slender relative to T0**	protein-DNA complex assembly, various other chromatin-related terms	anion channel activity
**T1 relative to T0**	RNA transport, translation regulation	endonuclease activity
**T6 relative to T0**	translation, signalling, calmodulin binding, hexose transport	cell adhesion
**T18 relative to T0**	oxido-reductase activity	cell signalling (cyclase activity)
**T48 relative to T0**	trans-splicing, translation	anion channel activity

Enrichment of particular GO groups were assessed, using a hypergeometric test, to determine whether the GO terms attached to each individual gene were overrepresented relative to all genes (and their associated GO terms) present on the arrays. The enrichment of GO terms in genes up- and down-regulated during different periods of differentiation are shown.

In order to determine if any genomic clustering was evident for co-regulated transcript groups during the differentiation programme we also analysed the chromosomal context of the 2166 genes identified as showing 2-fold expression differences at the 5% level in one or more of the comparisons: Slender *vs*. T0, T1 *vs*. T0, T6 *vs*. T0, and T48 *vs*. T0. These genes were plotted in physical order along each of the 11 chromosomes of the *T. brucei *genome and colour coded with respect to their up- or down-regulation according to the trinary code classes described above (Additional file 9). Supporting previous small-scale analyses of gene clustering for genes differentially regulated between different life-cycle stages, this revealed no extensive physical association of co-regulated gene clusters in the trypanosome genome. This provides further comprehensive evidence for regulation of the genome of *T. brucei *at the level of post-transcriptional operons.

### Analysis of stumpy-enriched transcripts by oligonucleotide motif scoring

We recently applied an oligonucleotide frequency scoring algorithm to identify nucleotide motifs statistically over-represented in the 3'UTR of procyclic form enriched transcripts in order to identify potential regulatory sequences [62]. The same approach was applied here to analyse the subset of transcripts predicted to be up-regulated in stumpy forms, these being searched for sequences statistically overrepresented when compared with either slender forms, or differentiating parasites. As in our previous analysis a training set derived from the 300 nt downstream of the stop codon of all predicted open reading frames on chromosome 1 and 2 (883 genes) of the *T. brucei *genome was generated. This provided frequency tables which were used to interrogate the 300 nt region downstream of the 40 transcripts predicted to be enriched in stumpy forms (i.e. those genes with a -1, -1, -1, -1, -1 trinary profile; Additional file 5) this being carried out using the Regulatory Sequence Analysis Tools (RSAT) web server [65]. Comparison of the two datasets identified two related hexanucleotide sequences in the stumpy enriched cohort (TCTTAC and TTCTTA) these being named stumpy motif 1 and stumpy motif 2 (SM1 and SM2). Of the cohort of 40 stumpy-enriched transcripts, SM1 was detected in 7/40 transcripts (17.5%) whereas SM2 was present in 12/40 (30%) of transcripts, with at least one of the motifs being present in 33% of stumpy-enriched transcripts. This contrasted with the frequency of these motifs in the 300 nt downstream of every gene predicted in the *T. brucei *genome database (a set of 11,008 genes) of 6.2% (SM1) or 11.5% (SM2), respectively.

**Figure 10:**
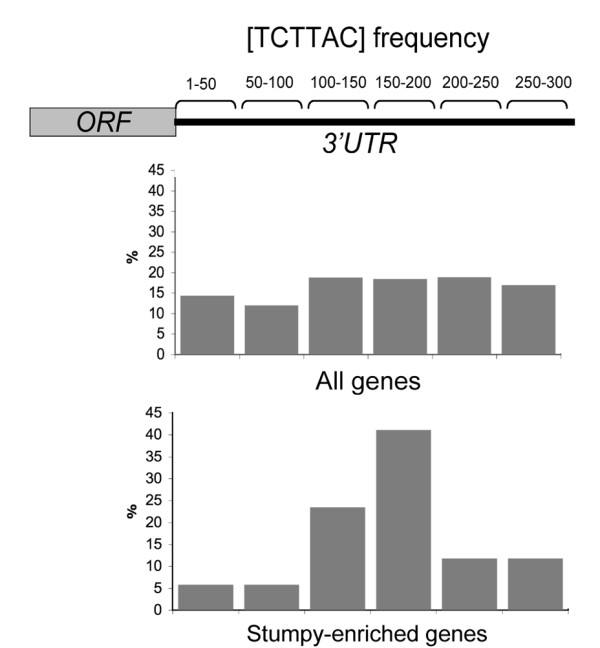
**Motif analysis for the regulation of transcripts predicted to be up-regulated in stumpy forms**. Oligonucleotide frequency analysis of the 300 nt downstream of genes identified as being up-regulated in stumpy forms with respect to other points in the differentiation programme. Two motifs were identified as being statistically overrepresented, of which SM1 (TCTTAC) was also elevated among the most strongly regulated genes in the stumpy expressed cohort. Positional analysis of the 3'UTR of those transcripts with this motif demonstrated a location bias 151-200 nt downstream of the stop codon of the associated gene. In contrast the position of the motif among genes not up-regulated in stumpy forms demonstrated no discernable positional bias.

Most interestingly, when we expanded the analysis to examine the 3'UTR of those genes with the highest overall probability of being enriched in stumpy forms when compared with slender forms (i.e. genes up-regulated in stumpy forms with an adjusted p value of between 0.01 and 0.06, this representing a cohort of 106 genes) we found that the context of the over-represented oligonucleotide sequences was not random (Figure 10). Thus, applying oligonucleotide frequency scoring to this gene set again identified the SM1 sequence (TCTTAC) as the most significantly over-represented motif, it being present at a frequency of 14% compared to 6.2% in the background dataset. However, when the position of the motif was determined in relation to the stop codon, we found that it was predominantly located 151-200 nt downstream of the stop codon, whereas there was no obvious positional enrichment of the motif in the 683 genes from the rest of the trypanosome genome that harbour SM1. We conclude that two related oligonucleotide motifs SM1 (TCTTAC) and SM2 (TTCTTA) are overrepresented in the 3'UTR of those mRNAs more abundant in stumpy forms and that SM1 shows a positional bias 150-200 nt downstream from the gene stop codon. This suggests that these sequences might represent context-specific regulatory motifs contributing to elevated gene expression in stumpy forms.

## Discussion

In this paper we describe an analysis of the mRNA expression profile of trypanosomes from two discrete bloodstream form stages of the parasite (slender and stumpy forms), as well as during the transition of the stumpy population to the procyclic life-cycle stage. Although previous analyses have compared either cultured or rodent derived bloodstream form parasites with cultured procyclic forms [37,38], our analysis represents the first comparison of in vivo derived pleomorphic slender cells with genetically identical stumpy forms, and a first analysis of the dynamic changes in mRNA profile that accompany the transition to procyclic forms. Previous analyses of the differentiation from stumpy to procyclic forms have established that this transition follows a defined and highly reproducible temporal programme, with progression through the major events of differentiation occurring with great synchrony in the population [33]. This provides a considerable power to the analysis of regulated mRNA changes because the observed changes at the population level can be used to direct infer changes at the individual cell level. Moreover, by tracking the progressive changes in mRNA abundance between time points in the differentiation programme, trends in the expression of individual or groups of transcripts can be identified, enabling regulated changes in expression to be distinguished from sample-specific variations.

Consistent with current consensus for the best optimisation of microarray analyses [66], our study focused on maximising the number of biological replicates, with 5 bio-replicates used for the stumpy and differentiating cell populations and 4 bio-replicates used for the pleomorphic slender populations. This allows both measurement and biological variability to be assessed and statistically tested, providing considerable statistical power over technical replicates alone. However, the consequence of this approach is that the ability to detect significantly regulated transcript groups is likely to be somewhat quenched due to the extent of biological variability, such that transcripts known to be differentially regulated may fall outwith the range of statistical significance. This is particularly the case when considering the analysis of stumpy and differentiating populations, which were derived after independent growth in individual mice over six days, likely generating considerable variability in many regulated mRNAs. Nonetheless, by detailed analysis of several cytological events of differentiation, the overall developmental progression of the distinct populations was found to be remarkably consistent enabling the identification of distinct regulated transcript profiles through the transformation process.

Our analyses focussed particularly on mRNAs up-regulated in the bloodstream stumpy form. These are the transmission stage of the trypanosome in the mammalian blood and have not been subject to extensive molecular analysis. A number of characteristics define stumpy forms cytologically: their characteristic morphology [27], cell-cycle arrest [67], resistance to proteolytic [29] and pH stress [30] and their capacity for differentiation in response to low concentrations of citrate/cis aconitate (CCA) when exposed to cold shock conditions [51,68]. Consistent with these characteristics, we observed that cell-cycle related transcripts were down-regulated in the stumpy forms (e.g. histone transcripts) whereas the PAD mRNAs (required for CCA reception) [51] were up-regulated. In addition to these changes we also observed by mRNA analysis and phenotypic validation that the lipid profile of stumpy forms was modified with respect to slender forms, with the expression of IPC representing either pre-adaptation for their differentiation to procyclic forms or early expression of this pathway. Indeed, individual analysis of the expression of each of the four sphingolipid synthase genes showed that one, *TbSLS1 *(Tb9.211.1030), exhibited particular developmental regulation, indicating that this gene may encode the *T. brucei *IPC synthase. Based on this prediction, a biochemical analysis has recently confirmed that *Tb*SLS1 has IPC synthase activity, functionally distinguishing it from the closely related *Tb*SLS4, which exhibits sphingomyelin/ethanolamine phosphorylceramide synthase activity [48] (Professor Jay Bangs, University of Wisconsin, USA; personal communication). Interestingly, squaline monooxygenase utilised in sterol biosynthesis was also up-regulated in stumpy forms. These changes in the lipid composition of stumpy forms perhaps provide explanation for the more robust characteristics of stumpy forms when compared to bloodstream slender forms [29,30] or demonstrate a requirement for rapid membrane rearrangement upon entry into the tsetse fly. Although a functional analysis of other stumpy-enriched transcripts is needed to dissect their role in stumpy form biology, several of the identified molecules are consistent with the characteristics of this life cycle stage. For example, the enhanced expression of two chloride channel proteins related to mammalian lysosomal chloride channel proteins may reflect the known elevated lysosomal activity of stumpy forms [50].

In an attempt to distinguish transcripts regulated as a pre-adaptation for the transformation to procyclic forms, we analysed mRNAs elevated in stumpy forms and 1 h into the differentiation programme. Unsurprisingly in these very transient expression profiles, some discrepancy with the validation assays was observed, possibly reflecting both normalisation bias and some non-systematic errors. Nonetheless, the analysis identified a relatively small subset of significantly elevated mRNAs, which comprised surface protein mRNAs, possible gene expression regulators, the aforementioned membrane lipid components and some metabolic enzymes. In all probability these will be a considerable under-representation of those molecules whose expression is enriched in stumpy cells because no account is taken of the differential translational efficiency of mRNAs whose overall abundance does not change significantly. Nonetheless, by bioinformatic comparison of these stumpy enriched mRNAs with the whole cohort of predicted genes in the trypanosome genome, enriched oligonucleotide sequences (SM1, SM2) could be identified in their 3'UTRs. These related motifs were statistically over-represented among stumpy mRNAs and one, SM1, exhibited a positional bias within the 3'UTR, being enriched 150-200 nt from the stop codon. Although the functional significance of over-represented oligonucleotide motifs requires experimental validation, the presence of a positional bias is not expected for a motif or motifs over-represented by chance alone and instead suggests a possible context-dependent function.

As well as focussing on stumpy-enriched mRNAs, we also analysed the dynamic changes in mRNA profiles during synchronous differentiation of stumpy forms to procyclic forms. Analysis of the resulting profiles particularly highlighted the increased abundance of mRNAs associated with the mechanisms of protein synthesis and translational control. This is not surprising: previous analyses of the polysomal profile of slender, stumpy and procyclic forms have established that stumpy forms are particularly quiescent, with metabolic labelling indicating that stumpy forms show only ~25% protein synthesis of bloodstream slender forms [44], TKS and KM, unpublished observations). After the rapid induction of mRNAs associated with translational processes, the parasites up-regulated mRNAs associated with cell proliferation, including genes required for DNA replication as well as structural components required for cell division. The kinetics of these molecular changes matched well the previously characterised cytological differentiation events, being induced after 6 h in differentiation conditions. Thereafter mRNAs associated with the metabolic changes that accompany differentiation were up-regulated. These changes indicate that there is rapid adaptation in terms of the mRNA profile as cells initiate differentiation to procyclic forms.

Although the tracking of known transcripts during the developmental events is informative and reassuring, the most interesting new information is likely to emerge from the analysis of genes which, although regulated, have no known function assigned or where distinct function cannot be assigned on the basis of sequence alone. Two examples that emerge from our analysis include the differential profile of sphingolipid synthesis genes described above and the observed transient elevation of histone deacetylase 2 during differentiation. Unlike the three other histone deacetylases in *T. brucei*, gene deletion of histone deacetylase 2 does not generate phenotype in bloodstream forms or during monomorphic cell differentiation [69]; however more detailed analysis of pleomorphs undergoing synchronous differentiation may uncover a subtle or unexpected role in development, as observed for these molecules in other systems [70]. Finally, although genes with similar expression profiles were not clustered in the genome, analysis of the regulatory motifs governing the co-expression of unlinked genes may allow cryptic regulatory motifs to be identified and post-transcriptional operons to be defined [71]. Hence, analysing genes with regulated expression profiles or which are co-regulated with other genes of known and unknown function should help to dissect the co-ordination of events necessary to generate a successfully differentiated procyclic form cell.

## Conclusion

The synchronous differentiation between bloodstream stumpy forms and procyclic forms in vitro has been exploited to identify regulated changes in gene expression during accessible developmental transitions of the African trypanosome. mRNA profiles were derived and analysed in the context of known biological markers for discrete steps in the differentiation programme and comparisons made between different samples at important points in its progression. Observed changes in gene expression have been verified by the analysis of known gene expression profiles or phenotypic analysis. The resulting data set will prove useful in analysing expression trends during transmission between bloodstream and procyclic forms of *Trypanosoma brucei*.

## Methods

### Trypanosomes and biological sample generation

*Trypanosoma brucei brucei *AnTat1.1 were used for all assays. For sample generation parasites were grown in cyclophosphamide treated mice and purified from host blood in PSG at 37°C via DEAE cellulose. Harvested parasites were then centrifuged at 2000 rpm in a clinical centrifuge, resuspended in pre-warmed (37°C) HMI-9 medium at approximately 1 × 10^8^/ml and settled for 60 minutes. Time 0 h time points were prepared by harvesting 2 ml of culture (2 × 10^7 ^parasites), the remaining 8 ml being added to 32 ml of SDM-79 medium containing 6 mM cis-aconitate and 10 mM glycerol. 10 ml aliquots were then taken at 1 h, 6 h, 18 h, with an additional 1 ml being harvested to prepare air dried smears, which were fixed in methanol at -20°C. At 18 h a further 10 ml of SDM79 was added to the remaining fluid (4-5 ml) and 10 ml of this harvested at 48 h for RNA preparation. Analyses of cell differentiation were carried out by immunofluorescence microscopy, as previously described [33], using antibodies specific for mouse anti-EP procyclin (1:500; Cedar Lane Laboratories) or rabbit anti-AnTat1.1 VSG (1:10,000; a kind gift of Professor Jay Bangs), with slides being counterstained with DAPI to allow visualisation of the cell nucleus and kinetoplast(s). Kinetoplast repositioning of 100 cells per sample was scored using ImageJ on a Zeiss Axioskop 2.

Pleomorphic slender cells were grown for 3 days in cyclophosphamide treated mice, then purified from host blood as for pleomorphic stumpy cells. The harvested parasites were then centrifuged at 2000 rpm in a clinical centrifuge and resuspended in HMI-9 medium, as above. Matching the treatment of stumpy forms, the slender cells were harvested after 1 h in HMI-9 and RNA prepared.

### RNA preparation

10 ml of cell suspension (~1 × 10^7 ^parasites) was centrifuged at 2000 rpm in a clinical centrifuge for 10 minutes, washed once for 1 minute with PSG and then the cell pellet lysed in 350 μl RLT buffer containing β-mercaptoethoanol, as specified in the "RNAeasy" RNA preparation protocol (QIAgen). RNA preparation was then completed according to the manufacturer's protocol, including the DNAse I treatment step.

### RNA analysis and array hybridization

Prior to use in microarray hybridizations, RNA samples were quality controlled using a Thermo Scientific NanoDrop™ 1000 Spectrophotometer to assess the quantity and then quality analysed on an Agilent RNA 6000 Nano chip (lab-on-a-chip), using a 2100 Agilent Bioanalyser. For target preparation, a primer containing poly dT and the T7 polymerase promoter was annealed to 500 ng of polyA+ RNA and reverse transcriptase used to synthesize the first and second strands of cDNA. Next, cRNA was synthesized from the double stranded cDNA using T7 RNA polymerase as specified by the manufacturer (low input linear amplification protocol; Agilent), this incorporating cyanine 3 (Cy3). Thereafter, 5 ug of the Cy3 target cRNAs were hybridised to the JCVI *Trypanosoma brucei *microarrays (version 3) for 18 hours at 60°C, washed, and scanned using an excitation wavelength of 532 nm and Cy3 detection filter in an Agilent microarray scanner (G2505B). Post hybridization and washing, microarray images were quantified using QuantArray software version 3 (Genomic Solutions). Northern blots were prepared and hybridized as described previously [62].

### Array normalisation and analysis

All array data analyses were performed in the R environment using Bioconductor packages ; . Briefly, array scans were background subtracted prior to QC analysis. Sub-standard arrays were removed from the analysis. The remainder were normalised within each array using printtiploess, and subsequently normalised across arrays using quantile normalisation. Data from control features were removed, and on-array duplicates averaged prior to linear model fitting and comparison of samples. Subsequently, empirical Bayesian analysis was applied (including p value adjustment for multiple testing, which controls for false discovery rate). The Bioconductor package limma was used (Smyth (2005) In:'Bioinformatics and Computational Biology Solutions using R and Bioconductor'. R. Gentleman, V. Carey, S. Dudoit, R. Irizarry, W. Huber (eds), Springer, New York, 2005). Gene lists, with accompanying statistical data, were generated for each comparison. Filtering of the data, whether by raw p value, adjusted p value, or fold change was as described within the text. Gene ontology (GO) enrichment analyses were carried out on appropriate subsets of the data, applying a hypergeometric test (available in the GOstats Bioconductor package). Up- and down-regulated loci were analysed separately across each of the three ontologies (BP: biological process; MF: molecular function; CC: cellular component). Array data has been uploaded to Gene Expression Omnibus with series accession ID GSE17026.

Oligonucleotide frequency scoring analysis was carried out essentially as described in [62].

### qRT-PCR

cDNA was made from 2 μg RNA using an oligo (dT)_15 _primer in a 20 μl total volume using the Reverse Transcription System (Promega) following the manufacturer's instructions, then diluted 1 in 5 with nuclease-free water. For qRT-PCR, 20 μl reactions were set up containing 5 μl cDNA in SYBR Green Master Mix (Roche) with 0.5 μM forward and reverse primers (these are listed in Table 3), these being validated by Amplify 3  and designed to amplify a 143-217 bp amplicon. Amplification was carried out in 96 well plates (Roche) in a LightCycler 480 machine comprising a 10 minute pre-incubation step at 95°C followed by 40 cycles of 95°C for 10 s, 55°C for 20 s, 72°C for 10 s with a single acquisition read at 82°C. A meltcurve analysis was performed from 65°C to 97°C with 2 acquisitions taken per °C, to test for primer dimer contamination. No-RT and no-template controls were run in each experiment and all wells were set up in duplicate. Relative quantification was calculated using the Pfaffl equation [72] using as a standard Tb10.389.0540, which was identified as being a stably expressed gene across all time points by array analysis.

**Table 3:** Primers used in qRT-PCR assays

Gene ID	Forward primer	Reverse primer
Tb10.26.0220	5' TCCAACCGATAACACGACAG 3'	5' CGATATGACCGACACGTCAC 3'
Tb927.7.7160	5' AGGCATCCATCGAGTACAGC 3'	5' TATCCTTCCGCAACACCTTC 3'
Tb10.6k15.3640	5' ACGGCCTCGTTGATACACTC 3'	5' CAACATTCCACCGACCATC 3'
Tb927.3.2710	5' GCAAGTCCATCACACAGGAG 3'	5' GAAGAGGCTACGGACACACC 3'
Tb927.1.4450	5' AGCAGCAGGTTATGGTGGAG 3'	5' ATACAACGATTCCGGTGAGC 3'
Tb10.389.0540	5' CCAGCCTTCTCAATCTCCAG 3'	5' GGCCACAGTTGGATAGCTTG 3'
Tb10.389.0650389.0650	5' ACGCTAGCACAACCAGAAGC 3'	5' GACCGACCAGGTCTTCTACG 3'

A list of all primers used to analyse gene expression profiles by qRT-PCR is shown with their associated Gene ID.

### Electrospray mass spectrometry analysis of parasite lipids

Total lipids from ~1 × 10^8 ^monomorphic bloodstream and stumpy trypanosomes were extracted by the method of Bligh and Dyer [73], dried under N_2, _and stored at 4°C. The lipid extracts were analyzed with a Micromass Quattro Ultima triple quadrupole mass spectrometer equipped with a nanoelectrospray source in both positive and negative ion modes. Tandem mass spectra (MS/MS) were obtained with collision offset energies as follows: 45 V, GPIno in negative ion mode, parent-ion scanning of m/z 241, as described previously [74]. Each spectrum encompassed at least 50 repetitive scans. Annotation of all phospholipids was also based upon comparison to their theoretical values and other ES-MS and ES-MS/MS analyses conducted on whole cell extracts (T. K. Smith, manuscript in preparation). Each spectrum encompassed at least 50 repetitive scans.

## Authors' contributions

SK carried out all trypanosome differentiation assays, analysed differentiation markers, prepared RNA samples and completed northern and qRT PCR experiments. KF carried out oligonucleotide frequency analysis and motif identification. AR performed microarray hybridisations, AI performed statistical normalisation and bioinformatics data analysis. TKS analysed the lipid composition of slender and stumpy form parasites. PG contributed to the study design. KM conceived the study and, with SK, analysed the data and wrote the manuscript, which was approved by all authors.

## Supplementary Material

Additional file 1 **Pairwise comparisons between slender and individual time-points for all genes represented on the microarrays**. Each column contains data with respect to each oligonucleotide represented on the array, with the parent Gene ID, product description, Protein ID, oligonucleotide sequence, oligonucleotide co-ordinates, signal intensity and statistical association provided for each. Annotations with respect to the presence of a signal peptide, transmembrane domain or GO classification are also detailed. Click here for file

Additional file 2 **Pairwise comparisons between different individual time-points for all genes represented on the microarrays**. Each column contains data with respect to each oligonucleotide represented on the array, with the parent Gene ID, product description, Protein ID, oligonucleotide sequence, oligonucleotide co-ordinates, signal intensity and statistical association provided for each. Annotations with respect to the presence of a signal peptide, transmembrane domain or GO classification are also detailed. Click here for file

Additional file 3 **Expression profile of all genes or those 407 genes exhibiting Log2 differential expression (P < 0.001) at one or more time points during differentiation**. Relative fold change in expression is given relative to stumpy forms (T0). Genes are sorted on the basis of relative differential between slender and stumpy forms, with genes up-regulated in slender forms with respect to stumpy forms having a positive value and genes down-regulated having a negative value. Associated statistical values for each comparison are available in Additional file 1 and Additional file 2. Click here for file

Additional file 4 **Genes exhibiting Log2 differential expression (adj P < 0.05 or adj P < 0.1) between pleomorphic slender and stumpy forms**. Sheet 1 shows genes up-regulated in slender forms with respect to stumpy forms; sheet 2 shows genes up-regulated in stumpy forms with respect to procyclic forms. Click here for file

Additional file 5 **Genes elevated in stumpy forms versus all other samples (trinary code profile -1,-1,-1,-1,-1) and genes elevated in stumpy cells and cells at T1 (i.e. 1 hour into the differentiation programme) (trinary code profile 0,-1,-1,-1,-1)**. Analysis of stumpy-enriched transcripts; only genes significant at the p < 0.05 level in one or more comparisons are considered, with the Log2 differential for each gene in each of the comparisons being assigned a trinary code based on whether the value was <-1 (code= -1), >1 (code= 1), or -1>x>1 (code= 0). Values are expressed relative to the expression of each gene in stumpy forms (T0) and are in the order T1 vs. T0, T6 vs. T0; T18 vs. T0; T48 vs. T0 and SL vs. T0. Click here for file

Additional file 6 **Summary of transient expression changes during differentiation to procyclic forms**. Genes were assigned to each temporal group based on their inclusion in the trinary profiles of transiently regulated genes. Click here for file

Additional file 7 **Expression profiles of genes exhibiting transient expression during differentiation**. Individual profiles of different transiently expressed groups as assigned to distinct trinary codes during the differentiation programme. Click here for file

Additional file 8 **Gene ontology analysis for genes temporally regulated during differentiation**. Genes on the array have been assigned annotation from any or all of the three gene ontologies (Biological process, BP; Molecular function, MF; cellular compartment, CC). The charts show the representation within distinct Gene ontology groups of significant genes (P < 0.05) within each comparison. Genes up-regulated and down-regulated are shown separately. Click here for file

Additional file 9 **Chromosomal location of the genes encoding regulated transcripts**. Physical clustering of genes exhibiting differential expression (P < 0.05) in the comparisons T1 vs. T0; T6 vs. T0; T18 vs. T0; T48 vs. T0 and SL vs. T0. The trinary codes for each gene significant at the 5% level were plotted in physical order along each chromosome. Blue denotes down-regulation, Red denotes up-regulation, white indicates 'no change'. No evidence of clustering was observed. Click here for file
