# Supplementary material for: Genome-wide expression profiling of in vivo-derived bloodstream parasite stages and dynamic analysis of mRNA alterations during synchronous differentiation in Trypanosoma brucei
Source: BMC Genomics. 2009 Sep 11;10:427. doi: 10.1186/1471-2164-10-427 (PMC2753553; doi:10.1186/1471-2164-10-427)

2–fold changes relative to T0,
Chr. 1, 136 sig. genes, 16 "unchanging", 35 different profiles

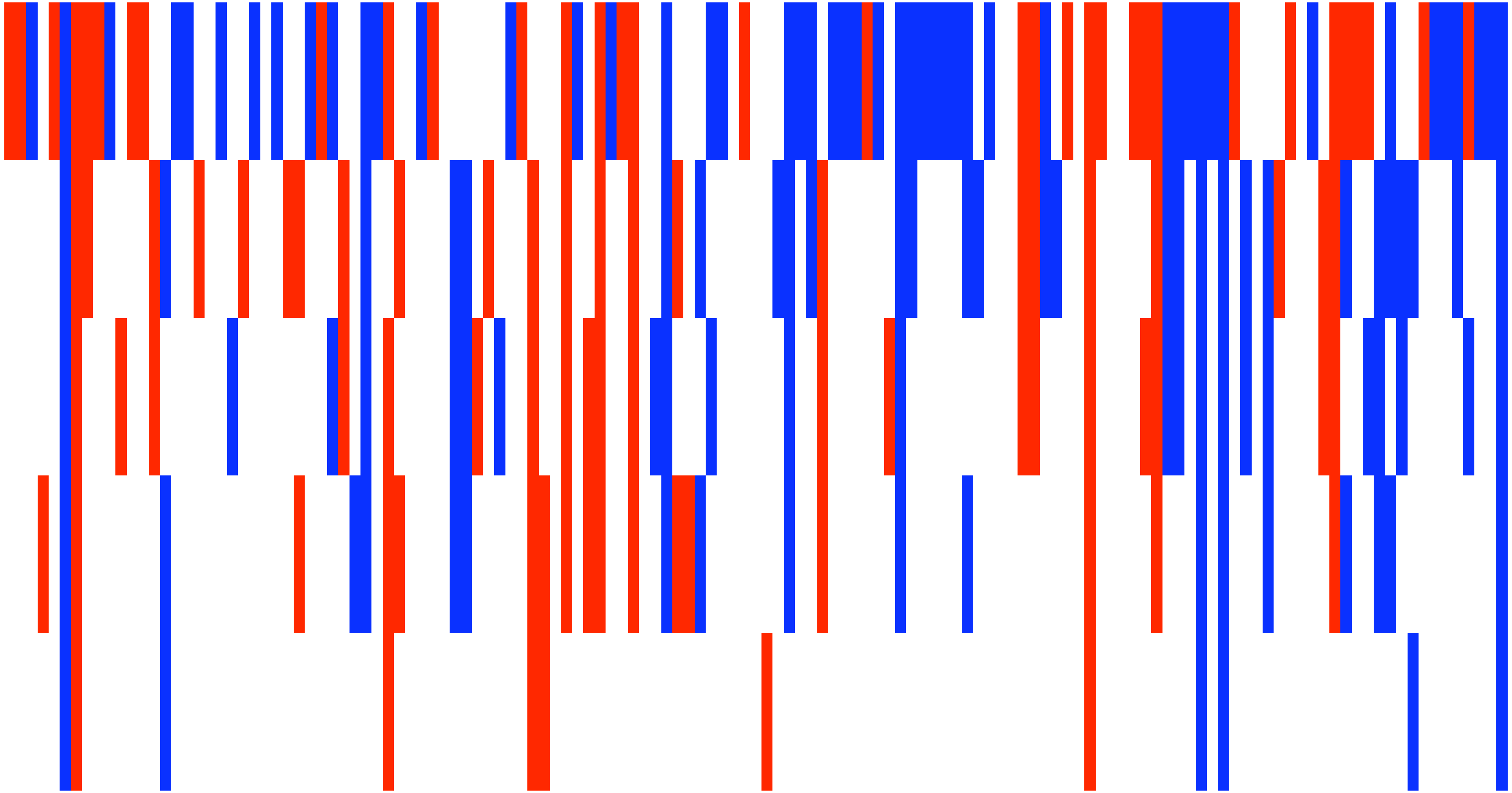

2–fold changes relative to T0, Chr. 2, 69 sig. genes, 9 "unchanging", 22 different profiles

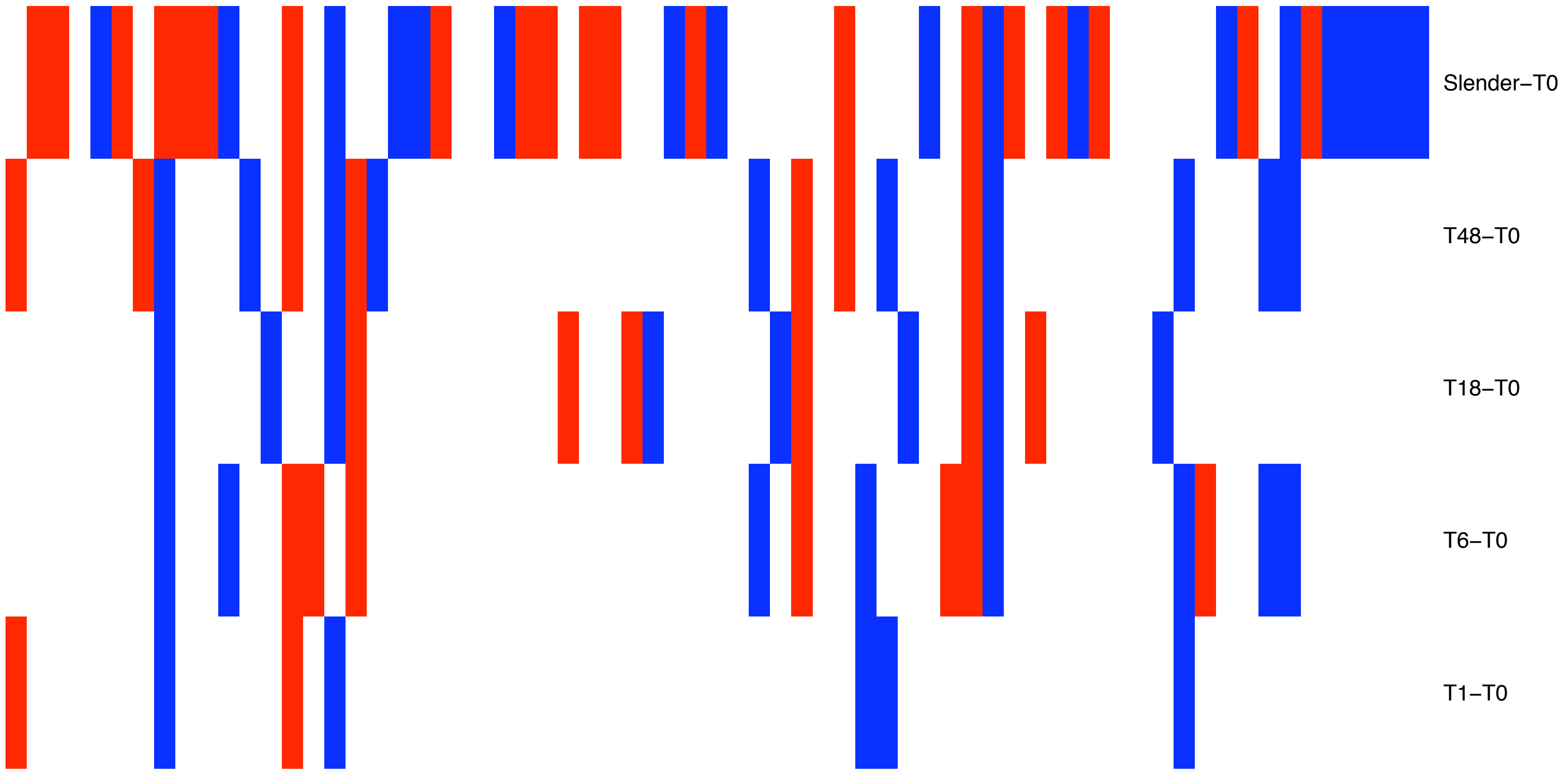

2–fold changes relative to T0,
Chr. 3, 102 sig. genes, 17 "unchanging", 31 different profiles

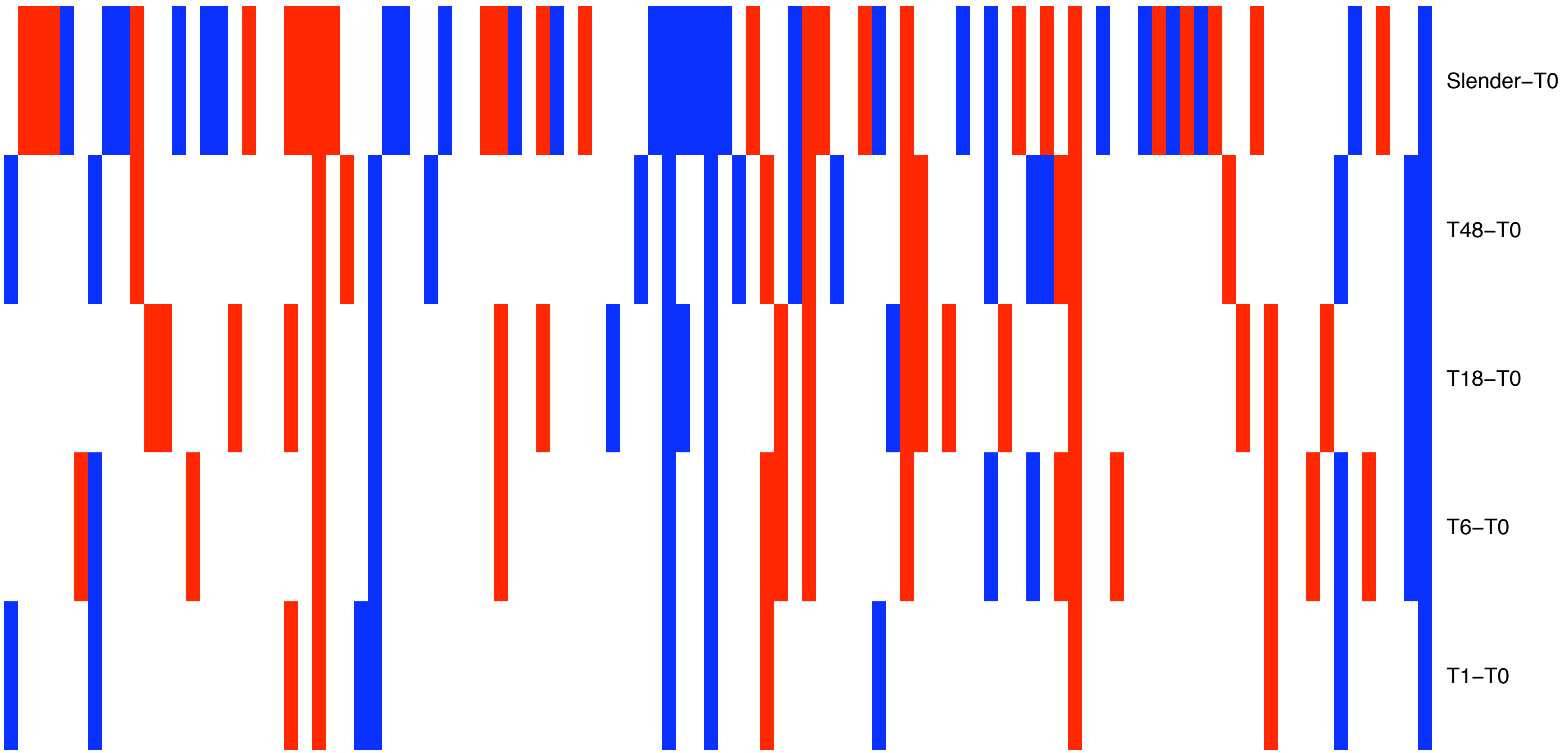

2–fold changes relative to T0,
Chr. 4, 118 sig. genes, 15 "unchanging", 31 different profiles

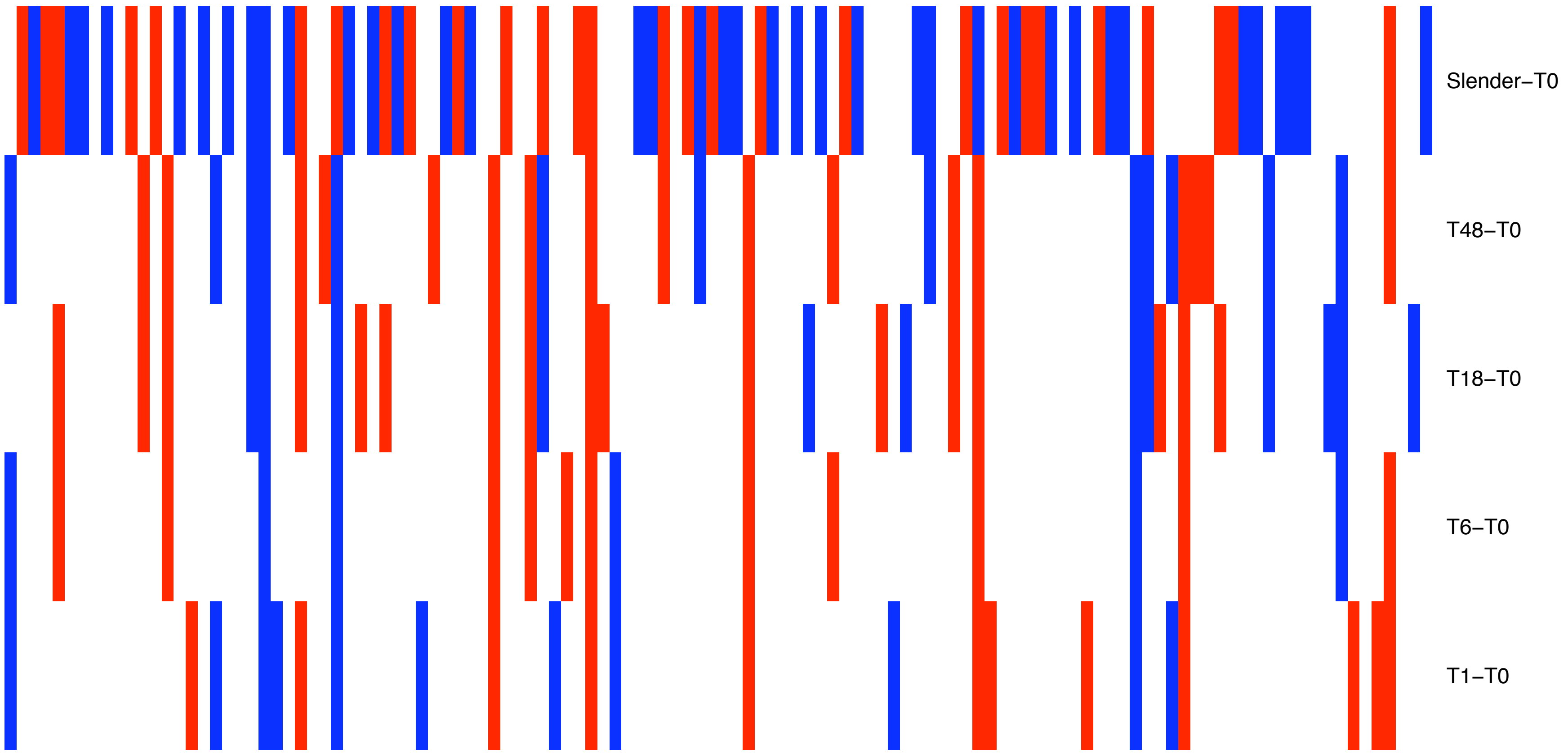

2–fold changes relative to T0,
Chr. 5, 90 sig. genes, 7 "unchanging", 26 different profiles

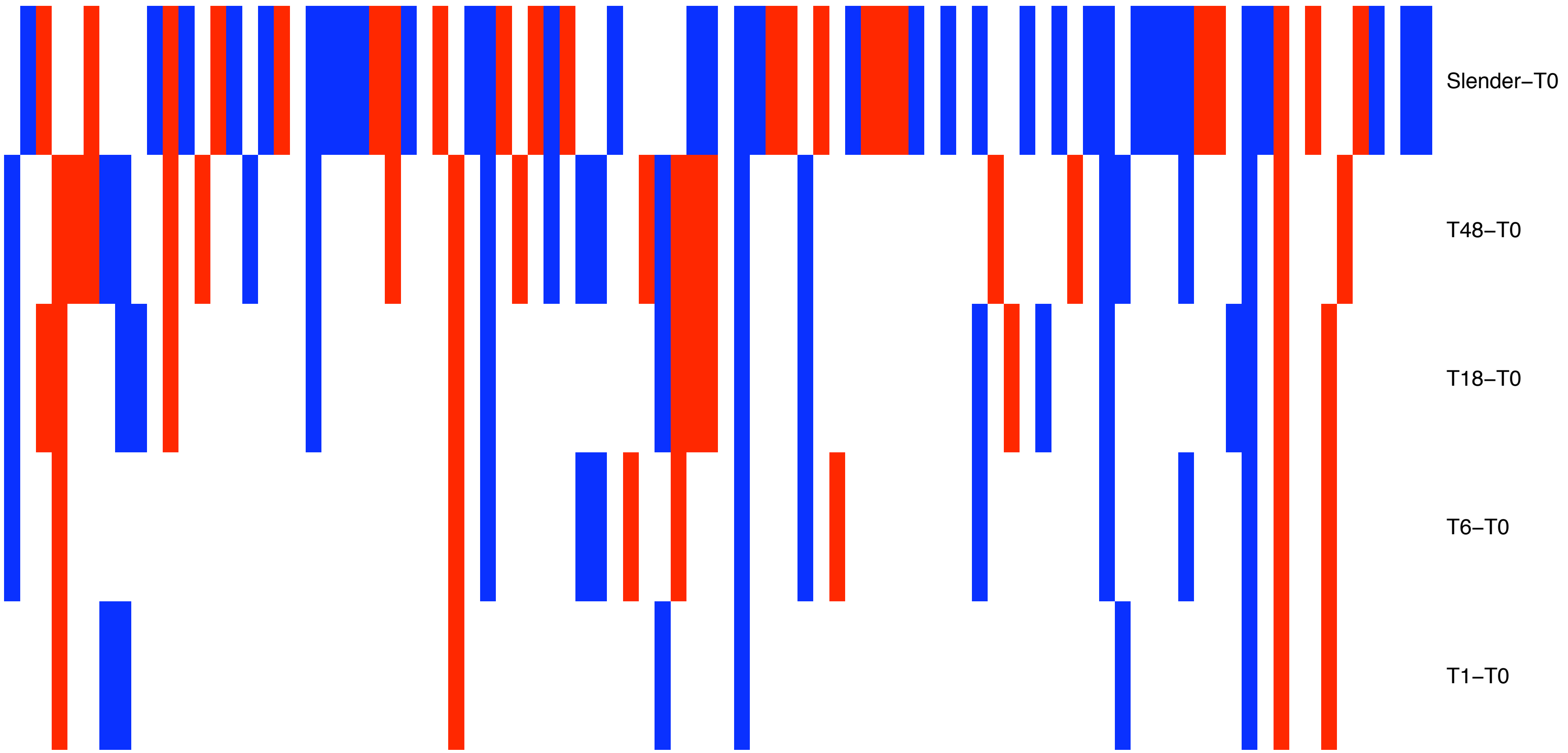

**2–fold changes relative to T0,
Chr. 6, 105 sig. genes, 13 "unchanging", 26 different profiles**

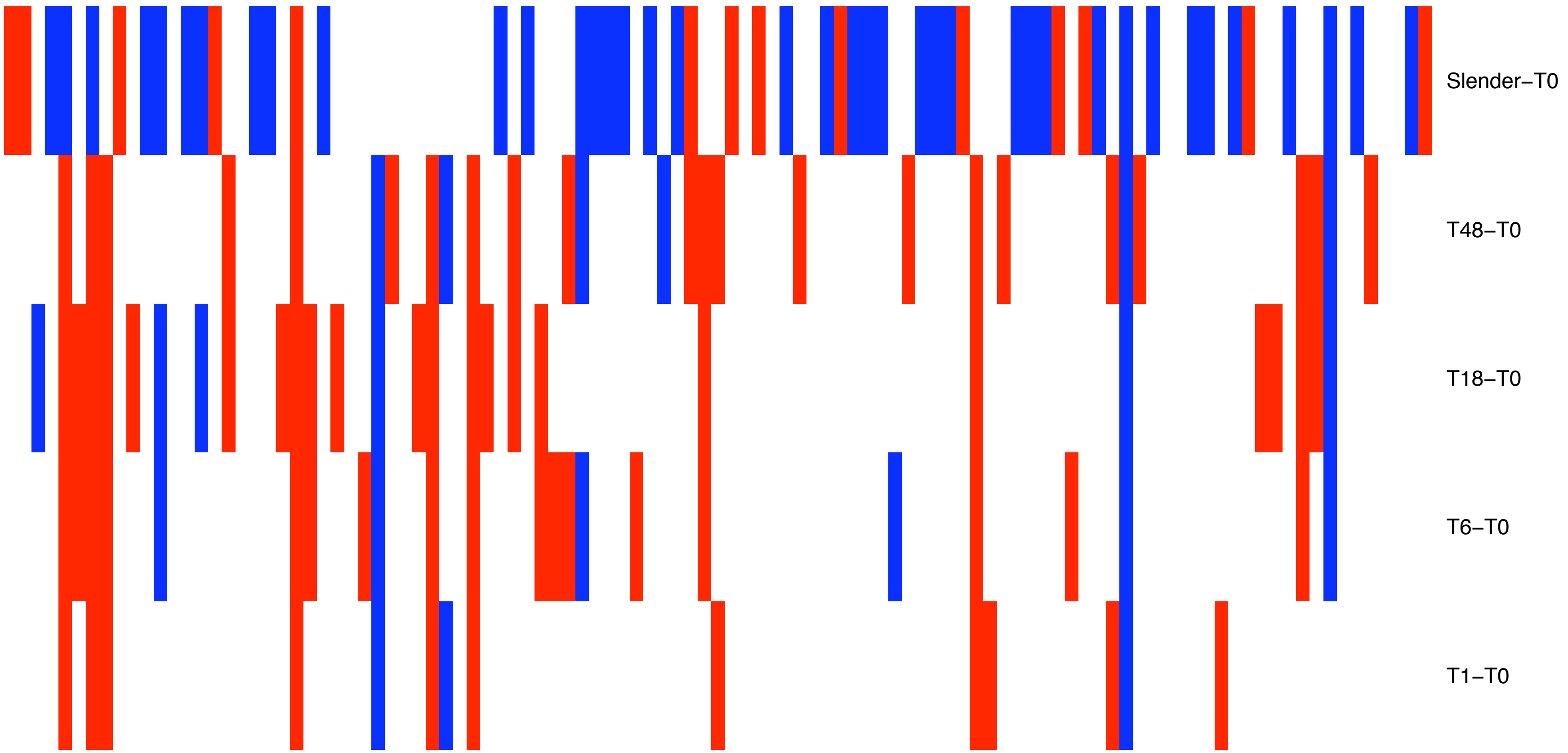

2–fold changes relative to T0, Chr. 7, 183 sig. genes, 23 "unchanging", 33 different profiles

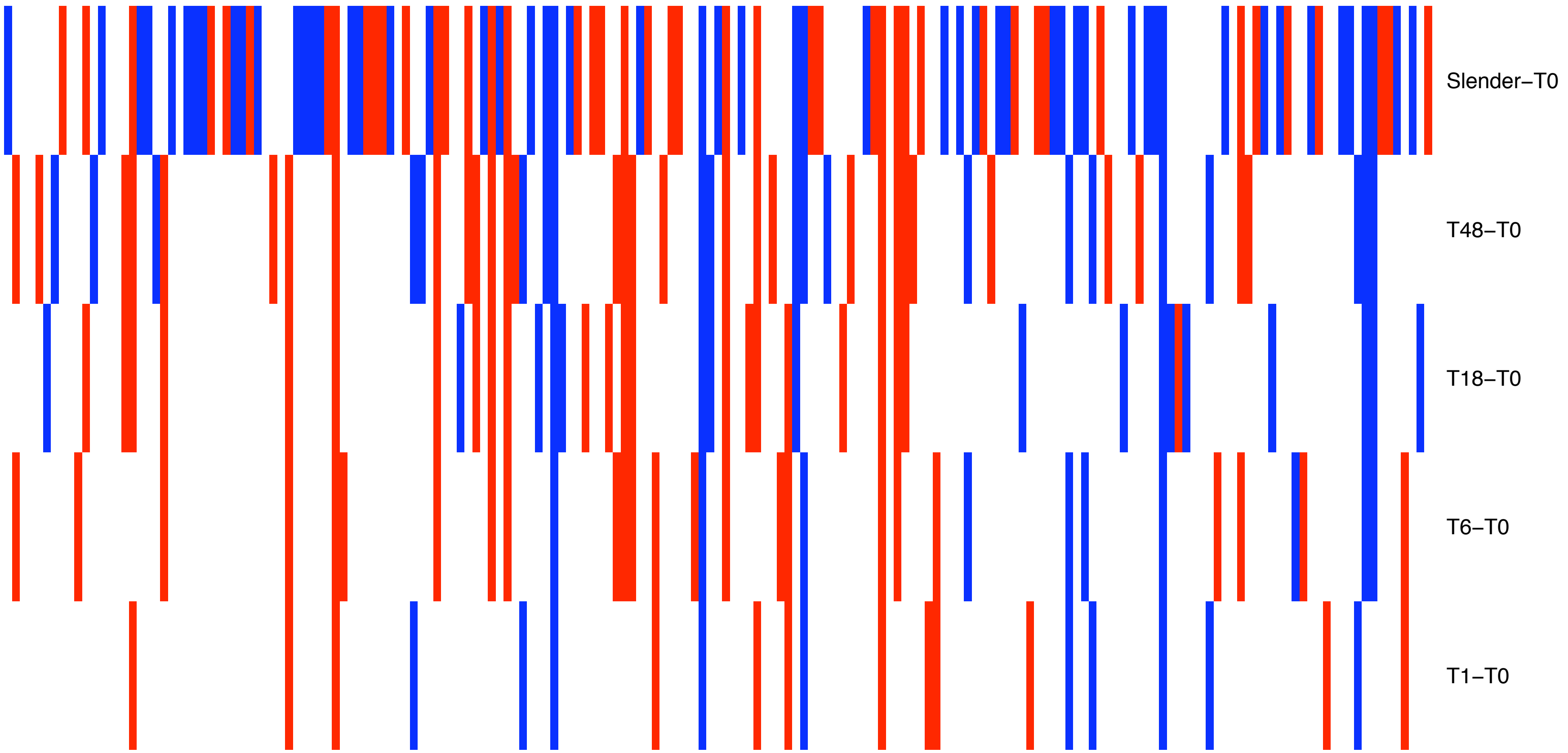

2–fold changes relative to T0,
Chr. 8, 176 sig. genes, 22 "unchanging", 36 different profiles

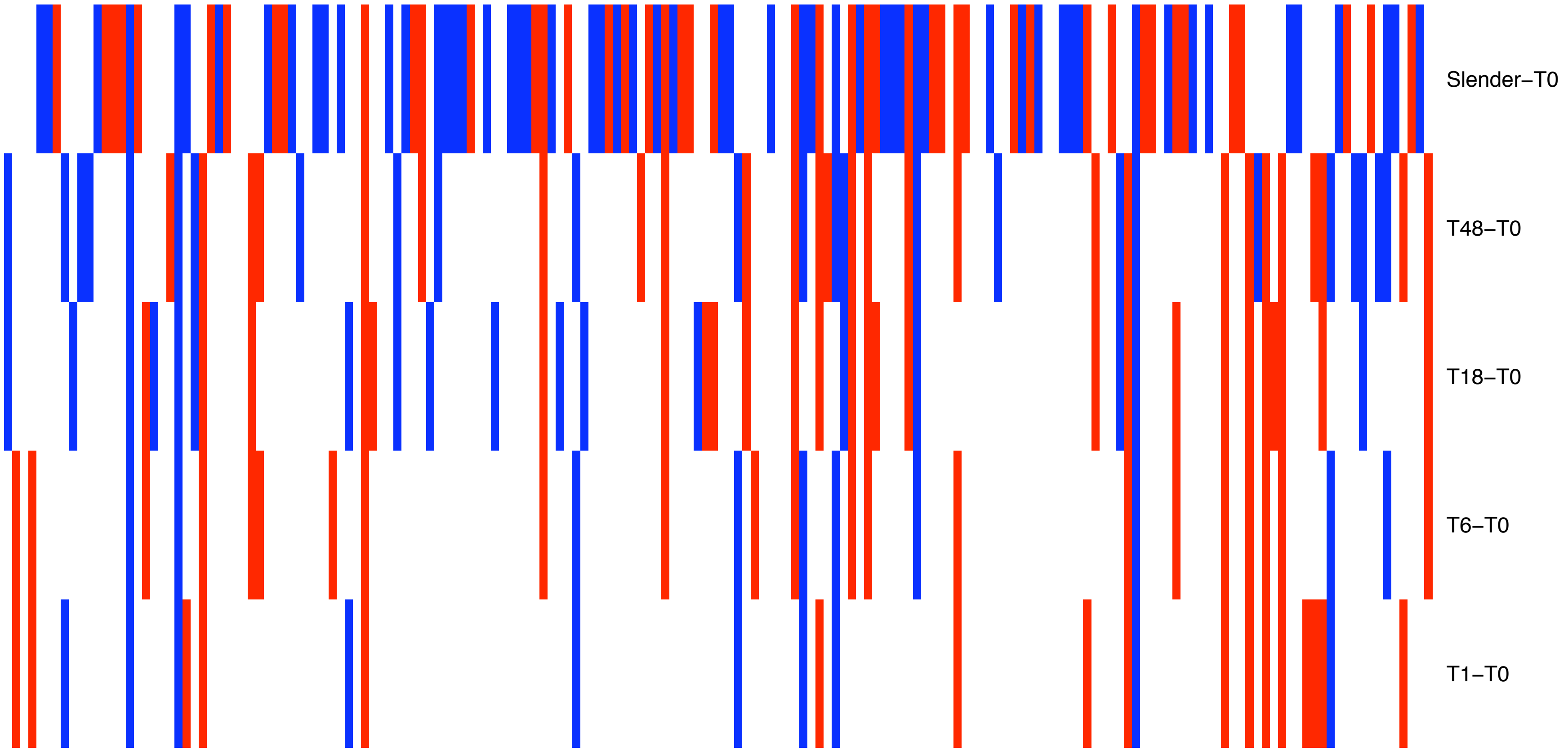

2–fold changes relative to T0,
Chr. 9, 347 sig. genes, 47 "unchanging", 50 different profiles

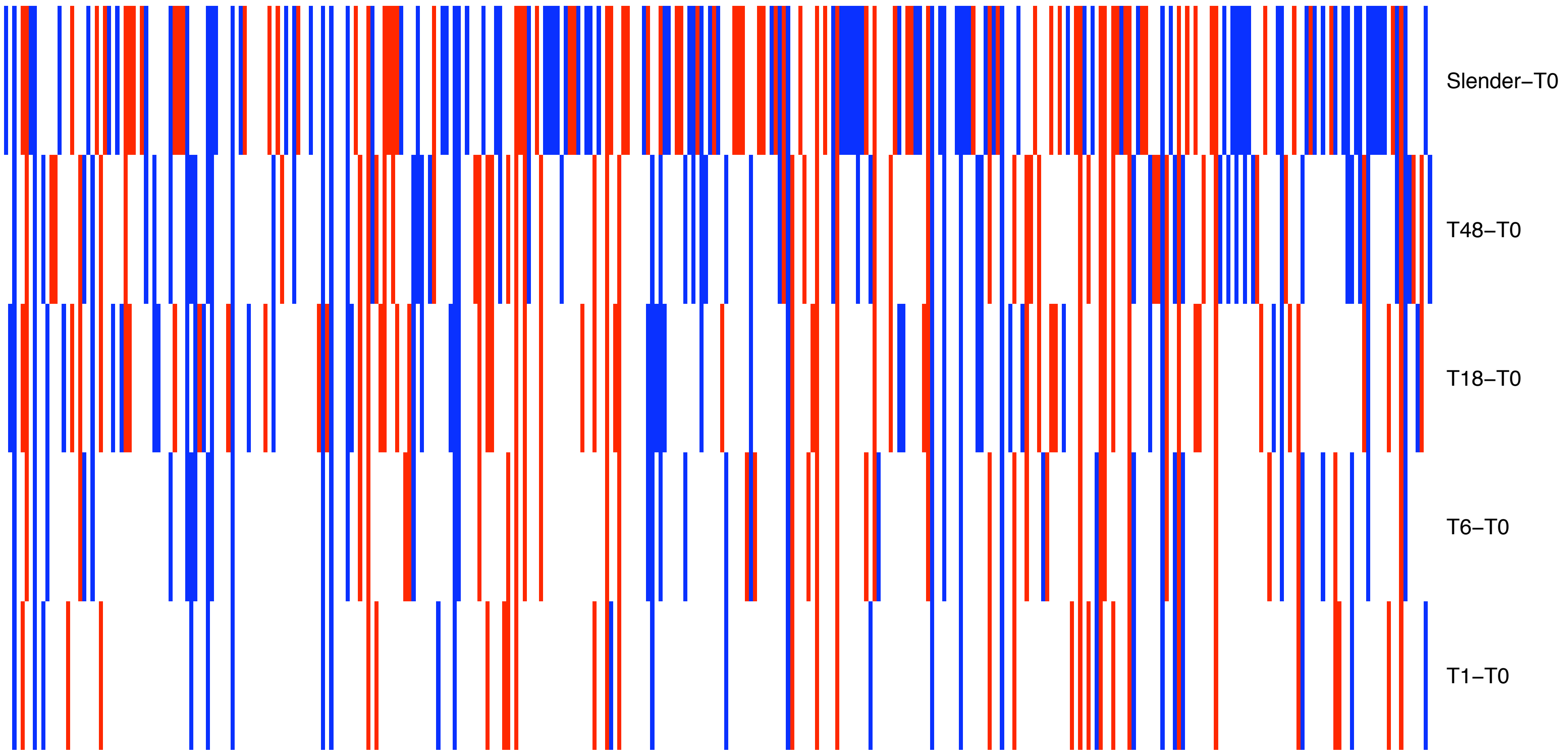

2–fold changes relative to T0,
Chr. 10, 369 sig. genes, 62 "unchanging", 43 different profiles

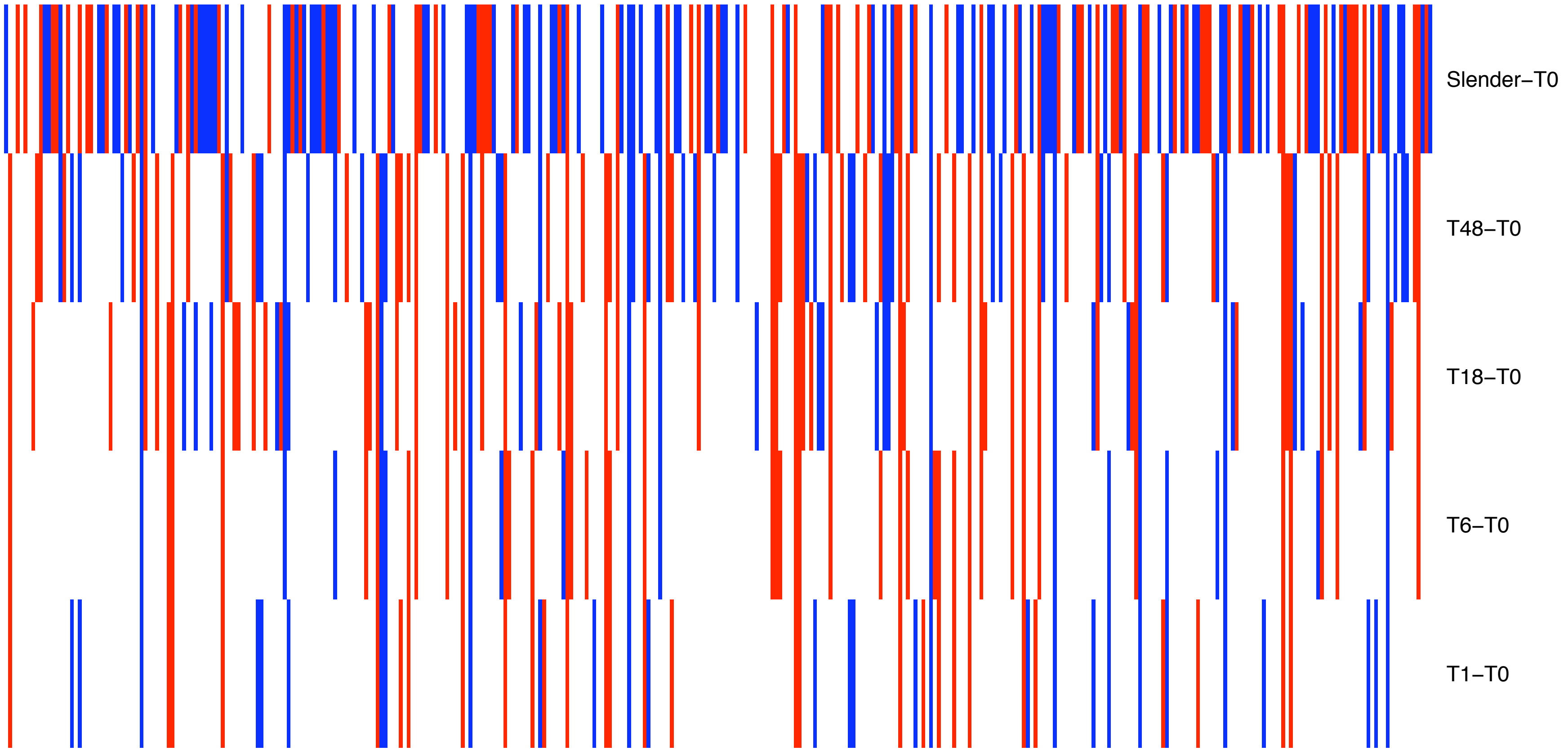

2–fold changes relative to T0,
Chr. 11, 385 sig. genes, 60 "unchanging", 48 different profiles

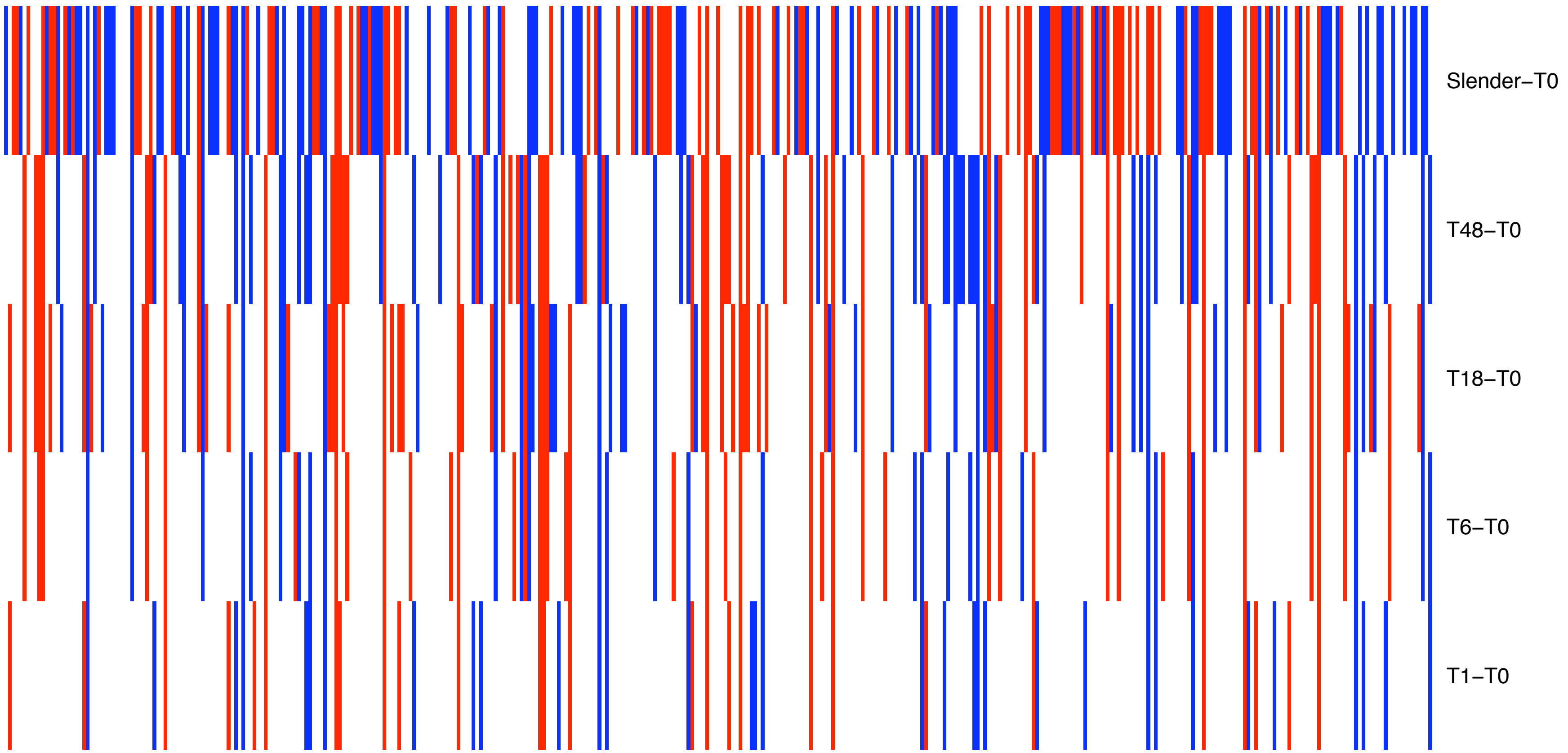

Supplement: Additional file 9 — Chromosomal location of the genes encoding regulated transcripts. Physical clustering of genes exhibiting differential expression (P < 0.05) in the comparisons T1 vs. T0; T6 vs. T0; T18 vs. T0; T48 vs. T0 and SL vs. T0. The trinary codes for each gene significant at the 5% level were plotted in physical order along each chromosome. Blue denotes down-regulation, Red denotes up-regulation, white indicates 'no change'. No evidence of clustering was observed. [file 1471-2164-10-427-S9.pdf]
